# HspBP1 is a dual function regulatory protein that controls both DNA repair and apoptosis in breast cancer cells

**DOI:** 10.1038/s41419-022-04766-0

**Published:** 2022-04-06

**Authors:** Cha Kyung Youn, Jung-Hee Lee, Gurusamy Hariharasudhan, Hong Beum Kim, Jeeho Kim, Sumi Lee, Sung-Chul Lim, Sang-Pil Yoon, Sang-Gon Park, In-Youb Chang, Ho Jin You

**Affiliations:** 1 DNA Damage Response Network Center, Chosun University School of Medicine, 375 Seosuk-dong, Gwangju, Republic of Korea; 2 Department of Cellular and Molecular Medicine, Chosun University School of Medicine, 375 Seosuk-dong, Gwangju, Republic of Korea; 3 Division of Natural Medical Sciences, Chosun University School of Medicine, 375 Seosuk-dong, Gwangju, Republic of Korea; 4 Department of Pathology, Chosun University School of Medicine, 375 Seosuk-dong, Gwangju, Republic of Korea; 5 Department of Anatomy, School of Medicine, Jeju National University, Jeju-Do, Republic of Korea; 6 Department of Hemato-oncology, Chosun University Hospital Internal Medicine, Gwangju, Republic of Korea; 7 Department of Anatomy, Chosun University School of Medicine, 375 Seosuk-dong, Gwangju, Republic of Korea; 8 Department of Pharmacology, Chosun University School of Medicine, 375 Seosuk-dong, Gwangju, Republic of Korea; 9 Present Address: Department of Meridian & AcupointᆞDiagnosis, College of Korean Medicine, Dongshin University, 67, Dongsindae-gil, Naju-si, Jeollanam-do Republic of Korea

**Keywords:** Homologous recombination, Breast cancer

## Abstract

The Hsp70-binding protein 1 (HspBP1) belongs to a family of co-chaperones that regulate Hsp70 activity and whose biological significance is not well understood. In the present study, we show that when HspBP1 is either knocked down or overexpressed in BRCA1-proficient breast cancer cells, there were profound changes in tumorigenesis, including anchorage-independent cell growth in vitro and in tumor formation in xenograft models. However, HspBP1 did not affect tumorigenic properties in BRCA1-deficient breast cancer cells. The mechanisms underlying HspBP1-induced tumor suppression were found to include interactions with BRCA1 and promotion of BRCA1-mediated homologous recombination DNA repair, suggesting that HspBP1 contributes to the suppression of breast cancer by regulating BRCA1 function and thereby maintaining genomic stability. Interestingly, independent of BRCA1 status, HspBP1 facilitates cell survival in response to ionizing radiation (IR) by interfering with the association of Hsp70 and apoptotic protease-activating factor-1. These findings suggest that decreased HspBP1 expression, a common occurrence in high-grade and metastatic breast cancers, leads to genomic instability and enables resistance to IR treatment.

## Introduction

Heat shock protein 70 (Hsp70) is the primary protein produced during cellular responses to various stresses and is involved in a plethora of folding processes [1]. Hsp70 binding protein 1 (HspBP1) binds to the ATPase domain of Hsp70 as a Nucleotide exchange factor (NEF) and inhibits ATPase activity [2–4]. In addition to this important function in protein quality control, Hsp70 acts as a powerful anti-apoptotic protein under multiple stresses due to its ability to relieve the toxicity of denatured and misfolded proteins that accumulate during stress [5]. The tumorigenic ability and poor therapeutic outcome associated with Hsp70 expression appear to be due to its role in conferring a survival advantage on tumor cells [6–11]. HspBP1 has a tumor-suppressive effect, which is attributed to its role in inhibiting Hsp70 pro-survival activity [12–14]. Since lower expression of HspBP1 in primary breast tissue is correlated to poor patient outcome [15], HspBP1 may play a protective role in tumorigenesis in breast cancer. However, the biological role of HspBP1 on tumorigenicity has remained poorly understood.

Breast cancer is the most common cancer among women, and the second common cause of cancer-related death in women. The incidence of breast cancer has increased by more than 50% over the last 25 years. Familial mutations in the BRCA1 tumor suppressor are a common cause of hereditary breast cancer [16]. BRCA1-deficient cancer cells exhibit impaired repair of double-strand breaks (DSBs) by the error-free mechanism of homologous recombination (HR) [17–19]. This defect leads to increasing genomic instability because these lesions are repaired by error-prone mutagenic pathways such as single-strand annealing and nonhomologous end joining (NHEJ). The ability of BRCA1 to maintain genomic integrity is thought to be important for its role in tumor suppressor [17, 20, 21].

Here, we show that HspBP1 plays an important role in inhibiting breast cancer tumorigenesis by promoting BRCA1-mediated HR repair. We also demonstrate that depletion of HspBP1 leads to increased resistance to ionizing radiation (IR) by preventing Hsp70 pro-apoptotic activity.

## Materials and methods

### Cell culture and treatment

Human osteosarcoma cell line U2OS and human cervix adenocarcinoma cell line HeLa were cultured in Dulbecco’s modified Eagle’s medium (Invitrogen, USA). Breast cancer cells (MCF-7, MDA-MB-231, and MDA-MB-436) and normal human fibroblast cell line (GM00637) were maintained in MEM medium (Invitrogen). All media were supplemented with 10% fetal bovine serum (FBS; Gibco, USA) and 1% penicillin/streptomycin antibiotic solution. The human normal breast cell line MCF-10A were maintained in DMEM/F12 (Gibco) supplemented with 5% horse serum (Gibco), 20 ng/ml EGF (Calbiochem, USA), 0.5 mg/ml hydrocortisone (Sigma-Aldrich, USA), 100 ng/ml Cholera Toxin (Sigma-Aldrich), 10 μg/ml insulin (Sigma-Aldrich), and 1% penicillin/streptomycin antibiotic solution. All cells were maintained in 5% CO2 in a humidified atmosphere at 37 °C. All cell lines except GM00637 cells were purchased from the American Type Culture Collection (ATCC, Manassas, VA, USA). GM00637 cells were obtained from the Coriell Cell Repository (Camden, NJ, USA). HCC1937 (BRCA1-defective breast cancer cells) and HCC1937BRCA1 cells (wild-type-reconstituted HCC1937 cells) were a gift from Ralph Scully (Harvard Medical School, Boston, MA, USA). All cell lines used in this study were tested for mycoplasma contamination, and all cells were confirmed to be mycoplasma-free. Irradiation were performed using a ^137^Cs source (Gammacell 3000 Elan irradiator, Best Theratronics).

### Antibodies

The antibodies used for immunoblot, immunoprecipitation, and immunofluorescence analysis are provided in Supplementary Table S1

### Plasmids and cell transfection

Human HspBP1 cDNA was amplified from GM00637 cells by reverse transcription PCR (RT-PCR) and cloned into the pEGFP-N3 mammalian expression vectors (Clontech, USA). The HA-BRCA1expretion vector was purchased from Addgene (Watertown, MA, USA). The HspBP1 shRNA-resistant mutant construct was generated by site-directed mutagenesis (Quikchange II Site-Directed Mutagenesis® kit, Agilent Technologies, Santa Clara, CA, USA) and confirmed by DNA sequencing. PCR primer sequences for site-directed mutagenesis were forward, 5’-ACAGCATGGATCGGAAGGATCCACCGGTCG-3’; reverse, 5’-CGACCGGTGGATCCTTCCGATCCATGCTGT-3’. pEGF-N3-HSPBP1 and pEGF-N3-HSPBP1 ΔMC expression vectors were generated as previously described [12]. Transfection of the cells with expression plasmids was performed with the use of the Lipofectamine 2000 reagent (Invitrogen). For establishment of the cells stably expressing Control-GFP or HspBP1-GFP, the cells were subjected to selection in culture medium supplemented with geneticin (400 μg/ml) after transfection, and individual resistant colonies were isolated to obtain cell clones.

### RNA interference (RNAi) and shRNA vector

Cells were transfected with siRNA using lipofectamine RNAiMax (Invitrogen) according to the manufacturer’s instructions. The HspBP1 siRNA sequences were HspBP1 siRNA#1, 5′-GTGCAGAAGCTCAAGGTCA(dTdT)-3′; HspBP1 siRNA#2, 5′-GAGCTGGAGTTCTGTGAAA(dTdT)-3′; HSP70 siRNA, 5′-GGACGAGUUUGAGCACAAG(dTdT)-3′. Scramble control siRNA was obtained from Bioneer (South Korea). For generation of stable HspBP1-depleted cell lines, oligonucleotides encoding the target sequences of HspBP1 were annealed and inserted into pSilencer2.1-U6-hygro vector (Thermo Scientific). The HspBP1 shRNA sequences were shRNA-sense, 5’-GATCC GTGCAGAAGCTCAAGGTCATTCAAGAGATGACCTTGAGCTTCTGCACTTTTTTGGAAA-3’; and antisense, 5’-AGCTTTTCCAAAAAAGTGCAGAAGCTCAAGGTCA TCTCTTGAATGACCTTGAGCTTCTGCACG-3’. U2OS, MCF-7, MDA-MB-231, and MDA-MB-436 cells were transfected with pSilencer2.1-U6-hygro control shRNA or pSilencer2.1-U6-hygro HspBP1 shRNA using lipofectamine 2000 (Invitrogen) and cultured in selection medium containing 500 μg/ml hygromycin B (Sigma-Aldrich) for 4-5 weeks. After selection, stable HspBP1 knockdown clones were confirmed by western blot analysis.

### Western blot and immunoprecipitation analysis

Cell extracts were prepared in RIPA buffer (50 mM Tris-HCl (pH 8.0), 150 mM NaCl, 1% Nonidet P-40, 0.5% sodium deoxycholate, 0.1% SDS) containing protease inhibitors (1 mM Na_2_VO_4_, 10 mM NaF, 2 mM PMSF, 5 μg/ml Leupeptin, 10 μg/ml Aprotinin, 1 μg/ml Pepstatin A) (Roche, Switzerland). Equal amounts of proteins were separated by SDS-PAGE followed by electrotransfer onto PVDF membranes (PALL Life Sciences, USA), Membranes were subsequently incubated with appropriate primary antibodies overnight at 4 °C, followed by incubation with peroxidase-conjugated secondary antibodies for 1 h at room temperature. The bands were visualized by using the ECL chemiluminescent detection system (iNtRON Biotechnology, South Korea). For immunoprecipitation of protein complexes, cell extracts were pre-cleared with protein G-Sepharose beads (GE Healthcare, USA) and incubated with appropriate antibodies. Immune complexes were then analyzed by immunoblotting.

### Comet assay

Neutral single-cell agarose-gel electrophoresis was performed for measurement of repair activity of DSBs. Cells were treated with 5 Gy of IR, followed by incubation in culture medium at 37 °C for the indicated times. TREVIGEN comet assay kit (TREVIGEN Instructions) was utilized to detect changed in repair activity. The stained slides with SYBR green (Lonza) were analyzed using a fluorescence microscope (Nikon) at × 400 magnification. The average comet tail moment was scored for 40–50 cells/slide using a computerized image analysis system (Komet 5.5; Andor Technology, Nottingham, UK).

### Homologous recombination assay

HR was measured using DR-GFP U2OS cells as described previously [22]. Briefly, DR-GFP U2OS cells were transfected with indicated siRNAs using Lipofectamine 2000 (Invtrogen). At 12 h after transfection, cells were transfected with pCBA-I-*Sce*I plasmid using Turbofect transfection reagent (Thermo Fisher Scientific). After 2~3 days, the percentage of GFP-positive cells was determined by flow cytometry (BD FACSCalibur, USA). For each analysis, 10,000 cells were processed and each experiment was repeated three times.

### Clonogenic ssay

Cells seeded in 6-well plates or 60 mm dishes (200 cells per well) were allowed to grow for 24 h before treatment with IR. They were then allowed to form colonies by incubation in drug-free medium for 7or 14 days. The resulting colonies were fixed with 70% ethanol and stained with 0.5% crystal violet and number of colonies were counted. The percentage of clonogenic survival was calculated as the ratio of the plating efficiency of treated cells compared to untreated cells. Results of clonogenic survival were presented as the mean value ± standard deviation for three independent experiments.

### CGH array and data analysis

Human fibroblast GM00637 cells and immortalized mouse embryonic fibroblasts (MEFs) were stably transfected with control or HspBP1 shRNA, using Lipofectamine 2000 (Invitrogen) and cultured in selection medium containing 0.2 mg/ml Hygromycin B (Sigma-Aldrich) for 2–3 weeks. After selection, stably HspBP1 knockdown clones were confirmed by western blot analysis. Genomic DNA from control shRNA-transfected GM00637 cells, HspBP1shRNA-transfected GM00637 cells, parent MEF, control shRNA-transfected MEF, and HspBP1 shRNA-transfected MEF was isolated using an AccuPrep^®^ Genomic DNA Extraction kit (Bioneer, Daejeon, South Korea) according to the manufacturer’s instructions. Array CGH analysis was performed using a NimbleGen CGH 12 × 135 microarray (Roche NimbleGen Inc., Madison, Wisconsin, USA) or a SurePrint G3 mouse genome CGH microarray (Agilent Technologies, Santa Clara, CA, USA). Briefly, human genomic DNA (1 μg) from control shRNA-transfected GM00637 cells and mouse genomic DNA (0.1 μg) from parent MEFs was labelled with Cy3-dCTP (as reference DNA). Human genomic DNA (1 μg) from HspBP1 shRNA-transfected GM00637 cells and mouse genomic DNA (0.1 μg) from control shRNA- or HspBP1 shRNA-transfected MEFs was labelled with Cy5-dCTP (as reference DNA) (as test DNA). The reference and test DNAs were cohybridize to the arrays, together with herring sperm DNA and washed. The hybridized array was scanned using Agilent’s DNA microarray scanner and Feature Extraction Software (Agilent Technology) was used to process the images. The log_2_ ratio of the fluorescence intensities of test to reference was calculated from background-subtracted median intensity values. A test:reference log_2_ ratio of zero implies no copy number change at that clone, positive and negative values indicates gain and loss of copy number, respectively.

### Chromosomal aberration analysis

Control and HspBP1-depleted U2OS cells were treated with or without 2 Gy IR. 24 h after IR, 100 ng/ml colcemid (Sigma-Aldrich) was added to arrest the cells in metaphase. 1 h after treatment, cell were harvested, gently resuspended in 40% of culture media for 10 min at 37 °C, and then fixed in methanol:acetic acid (3:1). After removal of supernatant, pellets were resuspended in fixative solution, dropped onto a glass slide and air-dried overnight. The slide was mounted in mounting medium with DAPI (Vectashield, USA). The metaphase images were captured using confocal microscope (Zeiss LSM 510 Meta; Carl Zeiss) and analyzed with image software ZEN (Carl Zeiss). At least 50 chromosomes were analyzed, and representative images were shown.

### Soft agar colony formation assay

Soft agar assays were performed in 6-well plates. The base layer of each well consisted of 2 ml (with a final concentration of 1x) medium and 0.6% low melting point agarose (Duchefa Biochemie, Netherland). Plates were chilled at 4 °C until solid. Next, 2 ml of growth agar layer was poured, consisting of 1 × 10^4^ cells suspended in 1x medium and 0.3% low-melting point agarose; plates were again chilled at 4 °C until the growth layer congealed. Further 1 ml of 1x medium without agarose was added on top of the growth layer. Cells were incubated at 37 °C with 5% CO_2_ for approximately 14–21 days, and total number of colonies were stained with 0.005% crystal violet (Sigma-Aldrich) and counted. Images were analyzed using an Olympus microscope (Olympus, Japan) and Image-Pro Plus 4.5 software (Media Cybernetics Inc., Rockville, MD, USA). Assays were repeated a total of three times.

### Tumor formation in nude mice

The mice used in this study were 6-week-old male BALB/c nude mice purchased from NARA Biotech (Seoul, South Korea). They were housed in our pathogen-free facility and handled in accordance with standard-use protocols and animal welfare regulations. MCF-7, MDA-MB-231, and MDA-MB-436 cells were harvested and resuspended in PBS. Thereafter, 3 × 10^6^ of control shRNA- and HspBP1 shRNA-transfected MCF-7 and MDA-MB-231 cells, 5 × 10^6^ of control GFP- and GFP-HspBP1-transfected MCF-7 and MDA-MB-231 cells, 5 × 10^6^ of control shRNA- and HspBP1 shRNA-transfected MDA-MB-436 cells, and 7 × 10^6^ of control GFP- and GFP-HspBP1-transfected MDA-MB-436 cells were injected subcutaneously into the left and right flanks of BALB/c nude mice. The mice were sacrificed after 40 days, and the tumors were excised. Once the tumors became visible, the tumor size was measured every 4 days using micrometer calipers. Tumor volumes were calculated using the following formula: volume = 0.5 *a* × *b*^2^, where *a* and *b* represent the larger and smaller tumor diameters, respectively. After 40 days of injection, mice were humanely sacrificed and the primary tumors were excised, immediately weighed, fixed in 10% Formalin solution (Sigma-Aldrich) and embedded in paraffin. All animal studies were reviewed and approved by the Institutional Animal Welfare and Use Committee.

### Immunofluorescence analysis

To visualize DNA damage foci, cells were seeded onto glass coverslips, treated with 5 Gy of IR, and incubated at 37 °C for indicated time points. Cells were then fixed with 4% paraformaldehyde for 10 min and ice-cold 98% methanol for 5 min, followed by permeabilization with 0.3% Triton X-100 for 15 min at room temperature. The coverslips were then washed three times with PBS, followed by freshly making blocking solution (5% bovine serum albumin in PBS) for 1 h at room temperature. Immunostaining with primary anti-BRCA1, anti-RAD51, anti-HspBP1, and anti-γ-H2AX antibodies was followed by further washed with PBS and then incubation with the appropriate Alexa Fluor 488, Alexa Fluor 594, or Alexa Fluor 647-conjugated secondary antibodies (Molecular Probes, USA). The coverslips were mounted in mounting solution with DAPI (Vectashield). Fluorescence images were taken under a confocal microscope (Zeiss LSM 510 Meta; Carl Zeiss, Germany) and analyzed with Zeiss ZEN Image software (Carl Zeiss). For foci quantification experiments, cells with > 5 foci were counted for positive cells and then percentage was calculated among at least 100 cells. The error bars represent standard deviation in three independent experiments.

### Immunohistochemistry

Hematoxylin/Eosin staining and immunohistochemistry (IHC) were performed on tissue microarray (TMA) of breast cancer. TMA from breast cancer samples of different grades and adjacent normal tissues were purchased from Biomax (Rockville, MD, USA) and Super Bio Chips (Seoul, South Korea). For IHC, heat-induced antigen retrieval was performed using 1x antigen retrieval buffer (pH 9.0) (Abcam) at 95 °C for 15 min. After quenching of endogenous peroxidase and blocking in 3% H_2_O_2_ solution, tissues were incubated with primary anti-Apaf1, anti-cytochrome C, and anti-HspBP1 antibodies overnight at 4 °C, followed by incubation with HRP-conjugated secondary antibody for 1 h at room temperature and then incubated for 2 min in DAB substrate. The slides were then counterstained by dropping Harris’s hematoxylin. HspBP1 immunoreactivity was determined by scoring for staining intensity (0, none; 1, weak; 2, moderate; 3, strong) and present of positive cells (0, < 5%; 1, 6–25%, 2, 26–50%; 3, 50–75%; 4, > 76%) and expressed as the product of both scores. The slides were analyzed by 2 independent pathologists.

### Ethics statement

All animal procedures were reviewed and approved by the Institutional Animal Welfare and Use Committee of Chosun University School of Medicine.

### Statistical analysis

All data were analyzed using Excel and Graphpad Prism software 6.0. Differences between two independent groups were tested with Student’s *t*-test. Correlations between different parameters were analyzed using Two-way ANOVA test with Bonferroni’s post-test. For the nonparametric statistical test, Mann-Whitney test was used. *P*-value of less than 0.05 was considered statistically significant and *P* values were indicated by asterisks as followed: *P < 0.05, **P < 0.01, and n.s. = non-significant. Error bars represent standard deviation (SD) of three independent experiments. All experiments were performed in triplicate, and repeated at least three times.

## Results

### HspBP1 prevents breast cancer tumorigenesis in a BRCA1-dependent manner both in vitro and in vivo

**Fig. 1:**
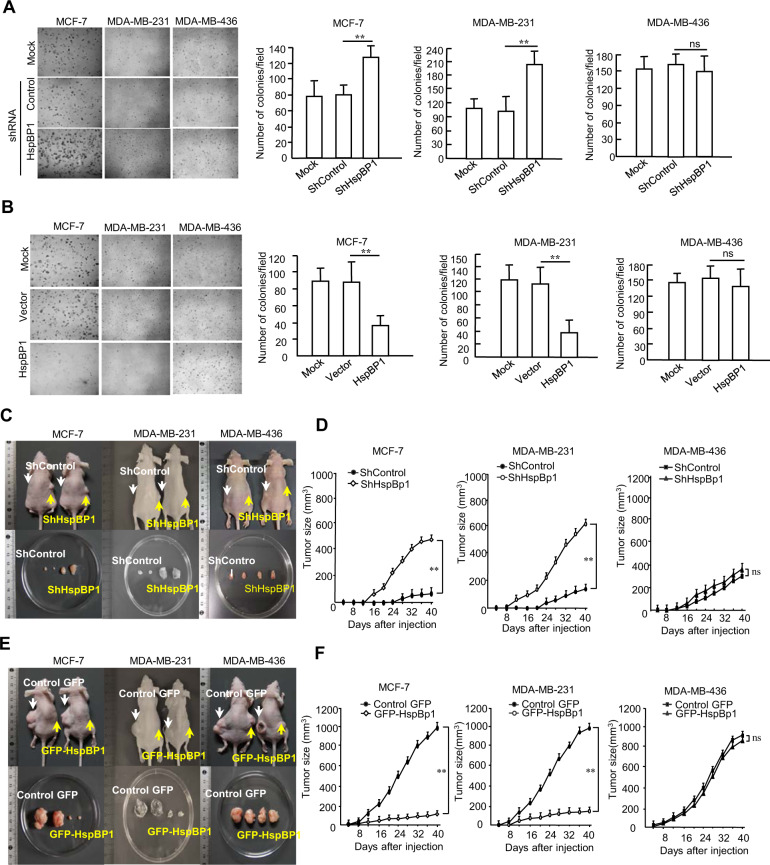
HspBP1 inhibits tumor growth of BRCA1-proficient breast cancer cells. **A** Anchorage-independent colony formation in soft agar of control and HspBP1-depleted BRCA1-proficient (MCF-7, MDA-MB-231) and BRCA1-deficient (MDA-MB-436) cells. Data represent the average cell number from 5 viewing fields and are shown as mean ± SD. **P < 0.01. ns, not significant, two-tailed Student’s *t*-test. **B** Anchorage-independent colony formation in soft agar of control and HspBP1-overexpressing MCF-7, MDA-MB-231 and MDA-MB-436 cells. Data represent the average cell number from 5 viewing fields and are shown as mean ± SD (*n* = 3). **P < 0.01. ns Not significant, two-tailed Student’s *t*-test. **C, D** Same cells described in (**A**) were injected into the left (control) and right flank of nude mice (*n* = 6). Photographs of representative mice and tumors (**C**) and growth curves of mammary tumors after implantation (**D**) are shown. Data are mean ± SD (*n* = 3). **P < 0.01. ns Not significant, two-way ANOVA. **E, F** Same cells described in (**B**) were inoculated subcutaneously into nude mice. Photographs of representative mice and tumors (**E**) and growth curves of mammary tumors after implantation (**F**) are shown. Data are shown as mean ± SD (*n* = 3). **P < 0.01. ns Not significant, two-way ANOVA.

To investigate whether the levels of HspBP1 expression affect tumorigenesis in breast cancer, we stably depleted HspBP1 in BRCA1-proficient (MCF-7 and MDA-MB-231) and BRCA1-deficient (MDA-MB-436) breast cancer cells using HspBP1-specific shRNAs (Fig. S1A) and stably overexpressed GFP-HspBP1 in each cell line (Fig. S1B). The HspBP1-depleted BRCA1-proficient cells showed an increase in anchorage-independent growth, whereas HspBP1-depleted BRCA1-deficient cells exhibited no difference from control cells (Fig. 1A). Moreover, only the HspBP1-overexpressing BRCA1-proficient cells had impaired colony formation in soft agar (Fig. 1B). When HspBP1-depleted cells were injected into mice, xenograft tumor growth and tumor size were significantly higher for BRCA1-proficient cells compared with controls over the 40-day time period of the experiments (Fig. 1C, D), but there was no difference for the BRCA1-deficient cells (Fig. 1C, D, third panel). However, when the HspBP1-overexpressing version of each cell line was injected into mice, both BRCA1-proficient cell lines led to markedly lower tumor outgrowth and size than the control, whereas the BRCA1-deficient cells did not (Fig. 1E, F). These results suggest that HspBP1 functions as a tumor suppressor in human breast cancer cells and the tumor suppressor function of HspBP1 seems to be dependent on BRCA1.

### HspBP1 regulates phosphorylation of BRCA1 and the subsequent recruitment of BRCA1 to regions of DNA damage

**Fig. 2:**
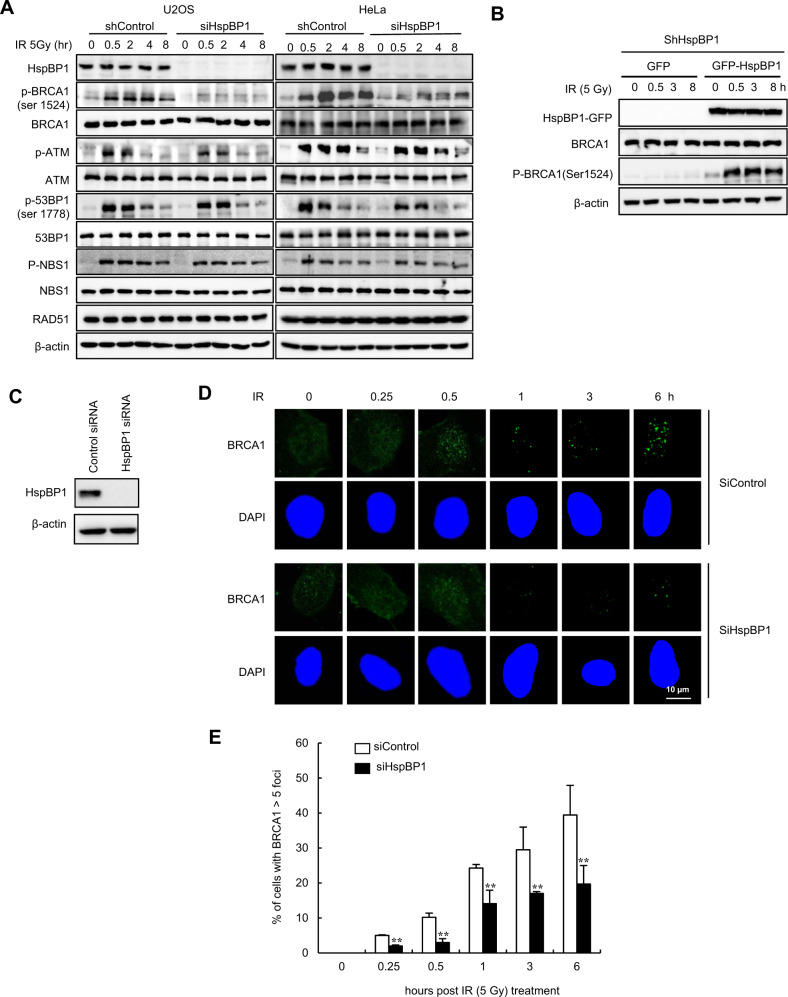
HspBP1 is important for BRCA1 phosphorylation and foci formation in response to IR. **A** Control and HspBP1-depleted U2OS and HeLa cells were either untreated or treated with 5 Gy ionizing radiation (IR). Whole cell lysates were prepared at the indicated time points and western blot analysis was performed using the indicated antibodies. **B** Stable HspBP1-depleted HeLa cells reconstituted with GFP-Mock or GFP-HspBP1 were either untreated or treated with 5 Gy of IR. Whole-cell lysates were prepared at the indicated time points and western blot analysis was performed using the indicated antibodies. **C** Levels of HspBP1 expression in control and HspBP1-depleted U2OS cells. **D, E** Control and HspBP1-depleted U2OS cells were either untreated or treated with 5 Gy IR, fixed at the indicated time points, and immunofluorescence analysis with antibodies against BRCA1 was carried out. Nuclei were stained with DAPI. Representative images (**D**) and percentage of cells populations that show more than 5 foci for BRCA1 foci (**E**) are shown. Data represent mean ± SD (*n* = 3), **P < 0.01, two-tailed Student’s t-test.

Since the tumor suppressive effect of HspBP1 is dependent on BRCA1, we predicted that HspBP1 might directly regulate BRCA1 function. To verify this, control and HspBP1 knockdown U2OS and HeLa cells were irradiated with IR and the levels of phosphorylated BRCA1, ATM, 53BP1, and NBS1 were measured at time points up through 8 h. DNA damage induced by IR resulted in increased phosphorylation of ATM, 53BP1, and NBS1 in control siRNA-transfected cells, as expected (Fig. 2A). Conversely, when HspBP1 was depleted in U2OS and HeLa cells, there was a marked reduction in IR-induced BRCA1 phosphorylation, but not ATM, 53BP1, and NBS1, suggesting that HspBP1 may specifically function upstream of BRCA1 in this pathway. Reconstitution of HspBP1 in BRCA1-depleted HeLa cells rescued IR-induced phosphorylation of BRCA1 to control levels (Fig. 2B), suggesting that the phenotype is not due to an off-target effect. We then monitored the dynamics of BRCA1 foci formation in response to IR in both control and HspBP1 knockdown U2OS cells (Fig. 2C). We found that BRCA1 foci formation was significantly reduced in HspBP1-depleted cells following IR treatment compared to control cells (Fig. 2D, E). Taken together, these data suggest that the HspBP1 contributes to BRCA1 phosphorylation and recruitment to sites of DNA damage in response to treatment with irradiation.

### HspBP1 is recruited to sites of DNA damage and interacts with BRCA1

**Fig. 3:**
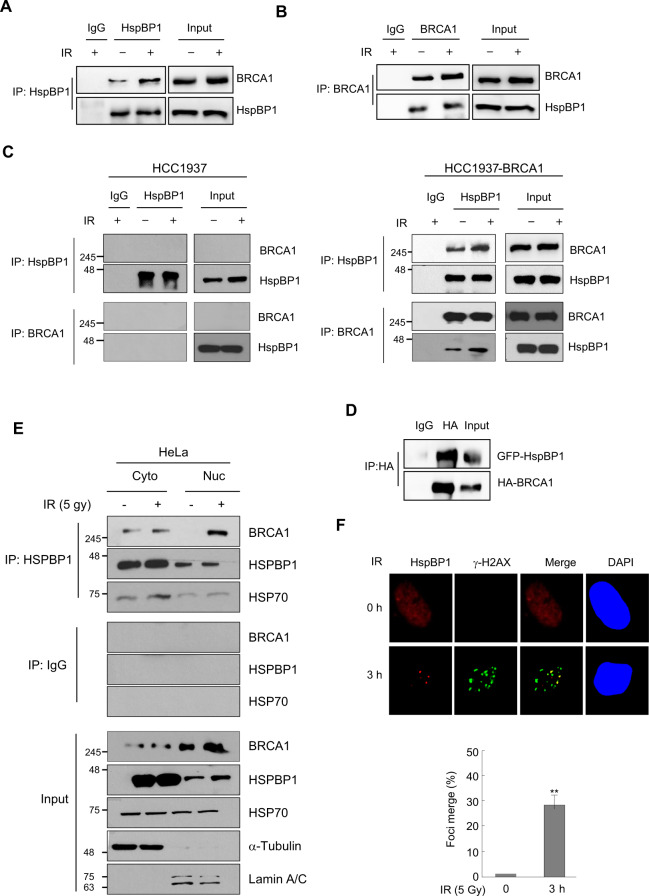
HspBP1 interacts with BRCA1 and is recruited to sites of DSBs. **A** HeLa cells were either untreated or treated with 5 Gy of IR for 3 h, after which whole-cell lysates were subjected to immunoprecipitation with anti-HspBP1 followed by western blotting with the indicated antibodies. **B** HeLa cells were prepared as in (**A**), and lysates were subjected to immunoprecipitation with anti-BRCA1 antibody followed by western blotting with the indicated antibodies. **C** HCC1937 and HCC1937-BRCA1 cells were treated with or without 5 Gy of IR. 3 h after IR treatment, whole-cell lysates were immunoprecipitated with anti-HspBP1 or anit-BRCA1 antibodies and subjected to western blot analysis with indicated antibodies. **D** HA-tagged BRCA1 was co-transfected with GFP-tagged HspBP1 into HEK293T cells and subjected to 5 Gy of IR treatment for 3 h, after which whole-cell lysates were subjected to immunoprecipitation with anti-HA antibody followed by western blotting with the indicated antibodies. **E** HeLa cells were treated with or without 5 Gy of IR. 3 h after IR treatment, lysates of the cytosolic faction and nuclear faction were immunoprecipitated with anti-HspBP1 antibody and subjected to western blot analysis with indicated antibodies. **F** HeLa cells were either untreated or treated with 5 Gy of IR and fixed at 3 h after irradiation. Localization of endogenous HspBP1 and γ-H2AX was detected using antibodies against HspBP1 (red) and γ-H2AX (green), and colocalization appears yellow in the merged image. Nuclei were stained with DAPI. Representative images and the percent colocalization between HspBP1 and γ-H2AX is shown in the graph. At least 100 cells were analyzed for each time point and data are presented as mean ± SD (*n* = 3), **P < 0.01, two-tailed Student’s *t*-test.

To elucidate the molecular mechanisms by which HspBP1 affects BRCA1 functioning, we first looked for direct interactions between HspBP1 and BRCA1. Immunoblotting analysis revealed that a substantial portion of endogenous HspBP1 and BRCA1 co-immunoprecipitated, and 5 Gy of IR treatment slightly increased the amount of BRCA1 bound to HspBP1 (Fig. 3A). A reciprocal assay using anti-BRCA1 antibody confirmed this interaction (Fig. 3B). We then tested the interaction between HspBP1 and BRCA1 in BRCA1 mutant breast cancer cell line HCC1937 and its derivative cell line reconstituted with wild type BRCA1 (HCC1937-BRCA1) [23]. HspBP1 and BRCA1 co-immunoprecipitated together from cell extracts of HCC1937-BRCA1 cells, but not HCC1937 cells (Fig. 3C). The presence of a BRCA1-HspBP1 complex in vivo was confirmed by demonstrating that full length HA-BRCA1 co-immunoprecipitated with GFP-HspBP1 in 293 T cells upon 5 Gy of IR exposure (Fig. 3D). Since BRCA1-mediated DNA repair occurs in the nucleus, cytosolic and nuclear fractions from HeLa cells treated with or without 5 Gy of IR were prepared and the interaction between HspBP1 and BRCA1 was tested. As shown in Fig. 3E, the interaction of HspBP1 with BRCA1 in the nuclear fraction was significantly increased in response to IR. On the other hand, the interaction between HspBP1 and BRCA1 was weak in the cytosolic fraction, and IR treatment did not significantly affect this interaction. Given the evidence for interactions between HspBP1 and BRCA1, we then examined whether HspBP1 is involved in double-strand break (DSB) repair by being recruited directly to sites of DNA damage. We used immunofluorescence staining to determine whether HspBP1 localizes at sites of DSB in response to IR treatment. Following exposure to IR, HspBP1 was indeed localized in discrete nuclear foci and co-localized with γ-H2AX (Fig. 3F), an indicator of DSB [24], suggesting that HspBP1 may play a direct role in DNA repair.

### HspBP1 depletion leads to impaired homologous recombination and an increase in chromosomal instability

**Fig. 4:**
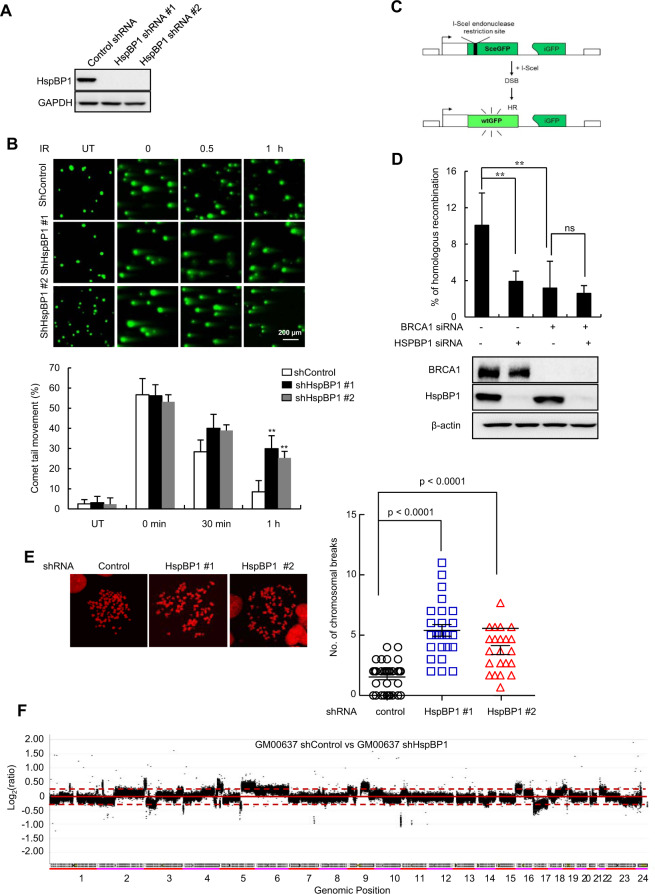
HspBP1 knockdown results in decreased DSB repair and increased chromosomal instability. **A** Immunoblot analysis of HspBP1 from a stable knockdown of HspBP1 using two different shRNAs in U2OS cells. **B** Control or HspBP1-depleted U2OS cells were either untreated (UT) or treated with 5 Gy of IR. At the indicated time points, cells were harvested for comet tail formation assays under neural conditions. Representative images and quantification of unrepaired DSBs are shown and data are presented as mean ± SD (*n* = 3), **P < 0.01, two-tailed Student’s *t*-test. **C** A schematic diagram of the fluorescence-based assay for measuring levels of HR-mediated DSB repair. **D** The efficiency of HR repair was measured by FACS analysis in DR-GFP-U2OS cells transfected with indicated combinations of siRNA. Levels of endogenous HspBP1 and BRCA1 were analyzed by western blotting and data are shown as mean ± SD (*n* = 3). **P < 0.01. ns, not significant, two-tailed Student’s t-test. **E** Control and HspBP1 knockdown U2OS cells were treated with 2 Gy of IR and chromosome aberrations were measured using metaphase chromosome spreads. Representative images and quantification of chromosome breaks are shown. *P* values between the indicated samples were calculated using a Mann-Whitney test. ns not significant. **F** Array CGH profiles of GM00637 cells transfected with control shRNA versus HspBP1 shRNA. Chromosomal regions above or below the dotted line indicate amplifications or deletions of genomic regions, respectively.

To look for downstream effects of the interaction between HspBP1 and BRCA1, we tested a HspBP1 knockdown for rates of DSB repair. HspBP1 was stably depleted in U2OS cells using two different HspBP1-specific shRNAs (Fig. 4A), and residual DSBs following IR exposure were measured using single-cell electrophoresis as an indicator of unrepaired DNA damage. In the absence of HspBP1, there were extensively longer comet tails than the control at 1 h after IR treatment (Fig. 4B), indicating that DSB repair is severely impaired. We then examined whether an HspBP1 depletion leads to a functional change in HR repair of DSBs. GFP-based chromosomal reporter assays in a stable cell line, DR-GFP-U2OS [25], were used to measure rates of HR (Fig. 4C) and the percentage of GFP-positive cells, indicating successful HR, in HspBP1-depleted cells was ~2.5-fold (p < 0.01) lower than in control cells (Fig. 4D), suggesting impaired HR function. To corroborate a functional link between HspBP1 and BRCA1 proteins, we simultaneously depleted both proteins and analyzed HR activity. Depletion of either HspBP1 or BRCA1 reduced HR efficiency to similar levels (Fig. 4D) and when both proteins were absent, there was no further reduction in rate (Fig. 4D, fourth lane) indicating that HspBp1 and BRCA1 function in same pathways.

The functions of HspBP1 relate to its well-established role as a Hsp70 nucleotide exchange factor. Therefore, we investigated whether HspBP1 contributes to the regulation of BRCA1-mediated DSB repair via its binding partner Hsp70. To this end, we generated a deletion mutant (aa 153 - 196 and aa 313 - 359) of HspBP1 (HspBp1-ΔMC) that cannot bind with Hsp70 as previous report [12]. Mock GFP, GFP-HspBP1-WT and GFP-HspBp1-ΔMC were then transfected into HspBP1-depleted HeLa cells, and the BRCA1 foci and DSB repair before and after IR exposure were checked. Indeed, reconstitution of HspBp1-ΔMC in HspBP1-depleted cells could rescue the IR-induced BRCA1 foci and DSB repair (Fig. S2A–E), suggesting that the role of HspBP1 on BRCA1-mediated DSB repair is independent of Hsp70.

Since failure to carry out HR is linked to chromosomal instability [21], we tested whether exposure to IR led to chromosomal abnormalities in HspBP1-depleted cells. Indeed, there were a significantly higher number of chromosomal breaks in HspBP1-depleted cells compared with control cells (Fig. 4E). We then performed array-comparative genome hybridization (array CGH) [26] and compared profiles between HspBP1-depleted and control untransformed normal human fibroblast GM00637 cells. A higher number of clonal amplifications and deletions in discrete regions were detected in HspBP1-depleted cells as compared with control cells (Figs. 4F, and S3A, B). Additionally, Similar results were also obtained in control and HapBP1-depleted MEFs (Fig. S3C, D). Together these findings suggest that a deficiency in HspBP1 leads to a decrease in HR-based DSB repair, resulting in a failure to maintain chromosome integrity.

### HspBP1 depletion impairs recruitment of Rad51 to sites of DNA breaks in BRCA1-proficient breast cells

**Fig. 5:**
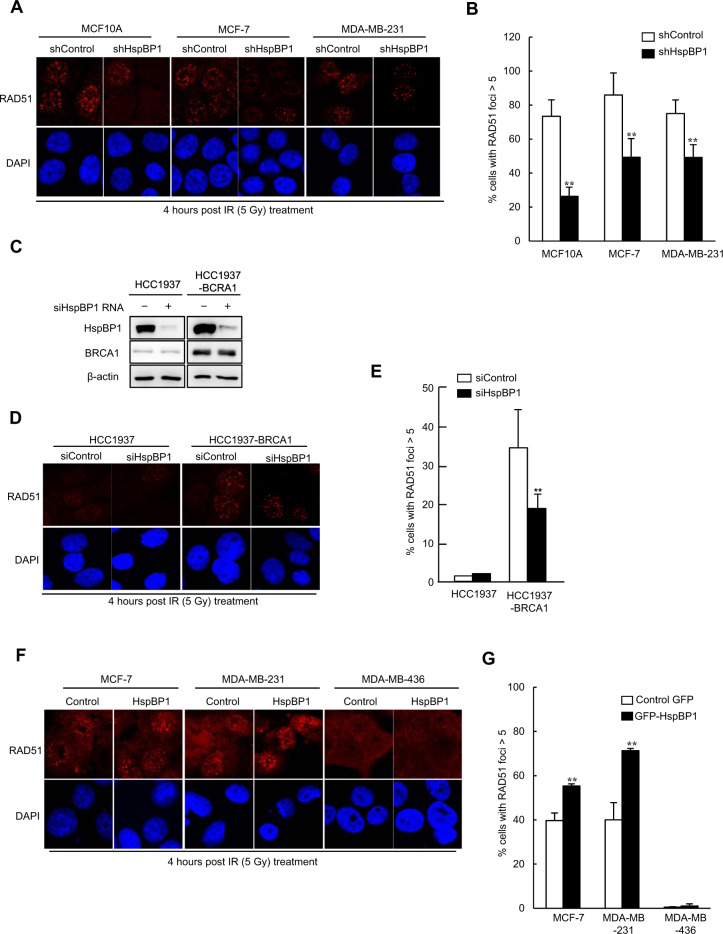
HspBP1 regulates Rad51 foci in response to IR in breast cancer cells. **A, B** HspBP1-depleted non-malignant breast cancer cells (MCF10A) or BRCA1-proficient breast cancer cells (MCF7, and MDC-MB-231) and corresponding control cell lines were irradiated with 5 Gy of IR and fixed at 1 h after irradiation. Immunofluorescence analysis with antibodies against Rad51 was then carried out. Nuclei were stained with DAPI. Representative images (**A**) and the percentage of cells with more than 5 foci for Rad51(**B**) are shown. Data represent mean ± SD (*n* = 3), **P < 0.01, two-tailed Student’s *t*-test. **C** The levels of HspBP1 in control and BRCA1-reconstituted HCC1937 cells transfected with either control or HspBP1 siRNA. **D, E** The levels of IR-induced Rad51 foci were measured in control and BRCA1-reconstituted HCC1937 cells transfected with either control or HspBP1 siRNA as described in (**A**). Representative images (**C**) and quantification (**D**) of Rad51 foci are shown. Data represent mean ± SD (*n* = 3), **P < 0.01, two-tailed Student’s *t*-test. **F, G** The levels of IR-induced Rad51 foci were measured in GFP-HspBP1-expressing MCF7, MDC-MB-231, MDA-MB-436 cells and the corresponding control cells as described in (**A**). Representative images (**F**) and quantification (**G**) of Rad51 foci are shown. Data represent mean ± SD (*n* = 3), **P < 0.01, two-tailed Student’s t-test.

Since BRCA1 is required for the recruitment of the central HR factor Rad51 to DBSs [27]. we looked for a role for HspBP1 in Rad51 foci formation in response to IR in breast cancer cells. HspBP1-depleted U2OS cells, HspBP1-depleted breast cancer MCF-7 and MDA-MB-231 cells, and non-malignant MCF10A cells (Fig. S4A) were tested for the ability to form IR-induced Rad51 foci. We observed that a stable knockdown of HspBP1 in BRCA1-proficient normal and malignant breast cells led to a significantly lower number of IR-induced Rad51 foci compared with control shRNA-transfected cells (Fig. 5A, B and Fig. S4B). We then tested the effect of HspBP1 depletion on Rad51 foci in HCC1937 and HCC1937-BRCA1 cells. HCC1937 cells displayed barely detectable IR-induced Rad51 foci, but reconstitution of these cells with wild-type BRCA1 restored the formation of Rad51 foci (Fig. 5D, E), as expected [28]. Importantly, the rescued Rad51 foci could be significantly reduced by expressing the HspBP1 siRNA in HCC1937-BRCA1 cells.

To further confirm whether the effect of HspBP1 on Rad51 foci is dependent on BRCA1, HspBP1 was overexpressed in both BRCA1-proficient and BRCA1-deficient breast cancer cells (Fig. S4C) and Rad51 foci were counted. We found that overexpression of HspBP1 in BRCA1-proficient MCF-7 and MDA-MB-231 cells promoted Rad51 foci formation in response to IR, whereas in BRCA1-deficient MDA MB-436 cells, overexpression of HspBP1 did not affect Rad51 foci (Fig. 5F, G). These results suggest that the effect of HspBP1 on Rad51 foci is highly dependent on BRCA1 function in normal and malignant breast cells.

### Depletion of HspBP1 causes cellular resistance to IR

**Fig. 6:**
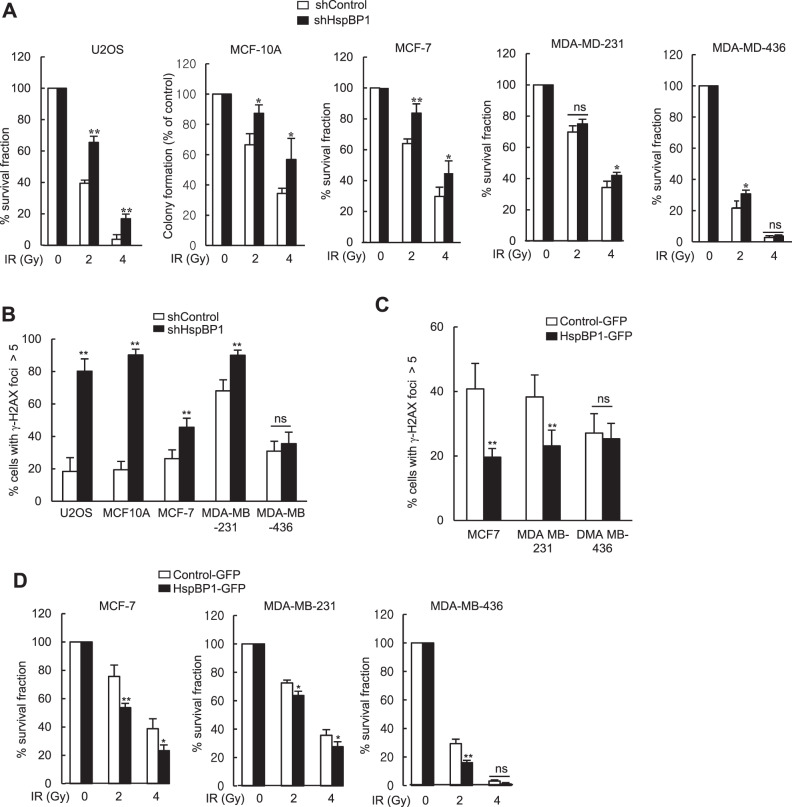
HspBP1 contributes to IR resistance. **A** Control and HspBp1-depleted U2OS, MCF-10A, MCF-7, MDA-MB-231, and MDA-MD-436 cells were either untreated or treated with the indicated doses of IR. The viability of untreated and treated cells were examined using the clonogenic survival assay. Results are shown as the mean ± SD (*n* = 3), **P < 0.01, *P < 0.05, ns, not significant. two-tailed Student’s *t*-test. **B** γ-H2AX foci of the same cells as described in (**A**). Cell were treated with 5 Gy of IR, fixed at 24 h, and immunostained using an anti-γ-H2AX antibody. The percentage of cells with more than 5 γ-H2AX foci is shown. Results are shown as the mean ± SD (*n* = 3), **P < 0.01, ns Not significant, two-tailed Student’s *t*-test. **C** Control and HspBP1-GFP-expressing MCF7, MDA-MB-231, and MDA-MB-436 cells were treated with 5 Gy of IR, fixed at 12 h after, and immunostained using antibody against γ-H2AX. The percentage of cells with more than 5 foci for γ-H2AX is shown. Results are shown as the mean ± SD (*n* = 3), **P < 0.01, ns Not significant, two-tailed Student’s *t*-test. **D** A clonogenic survival assay of the same cells described in (**C**). Cells were either untreated or treated with the indicated doses of IR. Results are shown as the mean ± SD (*n* = 3), **P < 0.01, *P < 0.05, ns Not significant, two-tailed Student’s *t*-test.

Because HspBP1 depletion leads to a reduction in the rate of repair of DBSs, which is important for cell survival following radiation injury, we speculated that HspBP1-deficient cells would be intrinsically sensitive to DNA damaging agents as well. To test this, we performed clonogenic survival assays in HspBP1-depleted and HspBP1-overexpressing cells treated with IR. Surprisingly, survival of HspBP1-depleted cells following IR, as measured by the number of colonies that grew, was higher than that of control cells (Fig. 6A) suggesting that the radioresistance conferred in the absence of HspBP1 occurred in all cells regardless of BRCA1 status. Of note, HspBP1-depleted U2OS, MCF10A, MCF-7, and MDA-MB-231 cells (BRCA1-proficient), but not HspBP1-depleted MDA-MB-436 cells (BRCA1-deficient), showed an increase in the number of unrepaired DSBs after IR-induced DNA damage as evidenced by the number of γ-H2AX foci that were retained as compared with the control (Figs. 6B, and S5A). However, even though HspBP1-overexpressing MCF-7 and MDA-MB-231 cells showed higher numbers of unrepaired DSBs in response to IR and HspBP1-overexpressing MDA-MB-436 cells did not (Figs. 6C, and S5B), all the HspBP1-overexpressing cell lines were more sensitive to IR treatment than control cells (Fig. 6D). Since depletion of HspBP1 increases anchorage-independent cell growth (Fig. 1A, B), the ability of HspBP1 for tumorigenesis may also affect cell survival in response to IR treatment. These results raise the possibility that in the absence of HspBP1, even if DSBs repair is compromised through impaired BRCA1 function, resistance to IR treatment is independent of BRCA1.

### HspBP1 depletion prevents IR-induced apoptosis by interfering with the association between Hsp70 and Apaf-1

**Fig. 7:**
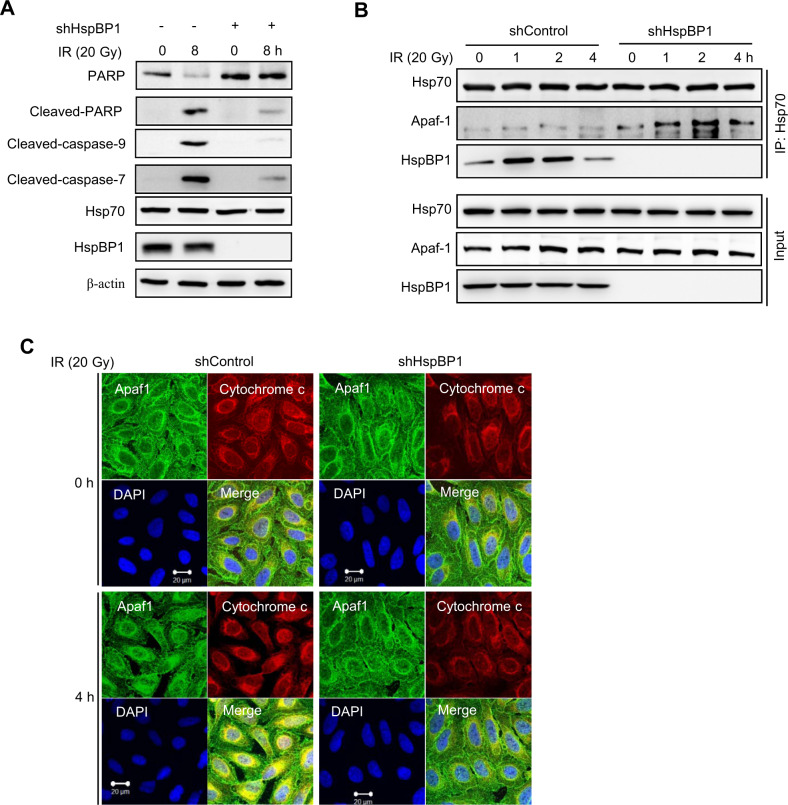
HspBp1 depletion inhibits IR-induced apoptosis. **A** Control and HspBP1-depleted cells were either untreated or treated with 20 Gy of IR for 8 h, after which whole cell lysates were subjected to western blotting using the indicated antibodies. **B** Control and HspBP1-depleted cells were either untreated or treated with 20 Gy of IR and lysed at the indicated time points. Whole-cell lysates were subjected to immunoprecipitation using anti-Hsp70 antibodies followed by immunoblotting using anti-Apaf-1, anti-Hsp70, and anti-HspBP1 antibodies. **C** Control and HspBP1-depleted cells were either untreated or treated with 20 Gy of IR for 4 h, after which they were fixed and immunostained using anti-Apaf-1 and anti-cytochrome c antibodies. Colocalization of Apaf-1 (green) and cytochrome c (red) is visible as yellow. A representative image is shown. Nuclei were stained with DAPI.

Our data suggest that although HspBP1 increases HR repair through interactions with BRCA1, depletion of HspBP1 promotes cellular resistance to IR. Therefore, HspBP1 may contribute to cell death through a BRCA1-independent manner. HspBP1 has been reported to antagonize the pro-survival activity and chaperone activity of Hsp70, which is a decisive negative regulator of apoptosis [2, 4, 12, 29, 30]. To determine the molecular basis underlying the resistance to DNA damage-induced cell death after knockdown of HspBP1, we tested whether HspBP1 knockdown has any effect on IR-induced apoptosis. As shown in Fig. 7A, cleaved PARP-1 fragment, a marker of apoptosis, was clearly observed in control cells at 8 h after IR irradiation. In contrast, when HspBP1 was depleted, levels of cleaved PARP-1 fragment were significantly lower, suggesting that HspBP1 prevents IR-induced apoptosis. To further define the role of HspBP1, we measured the level of activation of caspase-7 and caspase-9, two proteases that are essential in the intrinsic apoptosis pathway. We found that, upon IR exposure, there was significantly less cleavage of caspase-9 and caspase-7 into active fragments in HspBP1-depleted cells (Fig. 7A, third and fourth panel).

Hsp70 is known to interact directly with apoptosis protease-activating factor-1 (Apaf-1), inhibiting the recruitment of procaspase-9 to the apoptosome and thereby suppressing apoptosis [29–31]. Thus, HspBP1 may trigger DNA damage-induced apoptosis by modulating the binding of Apaf-1 to Hsp70. To test this hypothesis, we measured coimmunoprecipitation of Apaf-1 with Hsp70 over a time course of IR exposure, as shown in Fig. 7B. In control cells, we observed only weak binding of Apaf-1 to Hsp70 that increased just slightly at 4 hr after irradiation, but notably, there was significantly more binding in HspBP1-depleted cells, indicating that HspBP1 does indeed interfere with interactions between Hsp70 and Apaf-1.

Apaf-1 is activated by binding to cytochrome c, which is released from mitochondria in the early stage of apoptosis [32]. Therefore, we looked for a regulatory role for HspBP1 in this step of the pathway using immunofluorescence staining. Colocalization of Apaf-1 and cytochrome c in control cells was significantly increased in response to IR treatment, as expected (Fig. 7C), but was significantly lower in HspBP1-depleted cells, suggesting that HspBP1 influences this interaction. Taken all together, these data suggest that the presence of HspBP1 triggers IR-induced apoptosis through the Hsp70/Apaf-1/cytochrome c/caspase pathway.

### HspBP1 expression is inversely correlated with worsening grades of breast and ovarian cancers

**Fig. 8:**
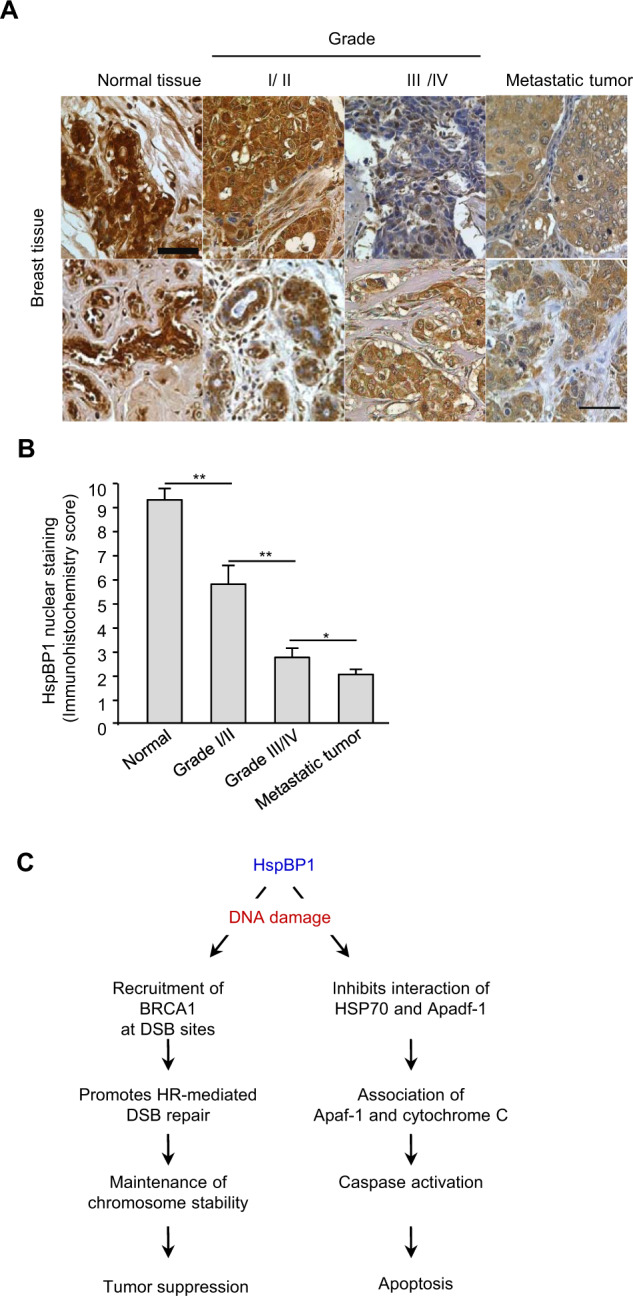
Expression of HspBP1 in normal and neoplastic breast tissues. **A** HapBP1 proteins in normal breast tissue, grade I/II, grade III/IV, and metastatic breast carcinoma are visualized using immunohistochemistry with an anti-HapBP1 antibody. The brown color indicates positive HspBP1 staining. Nuclei are stained with DAPI (blue). **B** Nuclear immunoreactivity for HspBP1 was blind-assessed and quantified by scoring for staining intensity and percent positive cells (see Materials and Methods) in tumor section of grade I/II, III/IV, metastasis, and normal tissue sections from the breast tissue microarray (total 85 samples). Results are shown as the mean ± SD (*n* = 3), **P < 0.01, *P < 0.05. **C** A schematic representing the role of HspBP1 in tumor suppression and apoptosis. See text for details.

HspBP1 levels are higher in breast cancer tissue compared to normal adjacent tissues, however, it is interesting to note that HspBP1 levels are significantly lower in patients with advanced-stage cancer and with metastasis to auxiliary lymph nodes [15]. Given that HspBP1, in association with BRCA1, functions within the nucleus to maintain genome stability and is thought to be important for BRCA1 tumor suppression, we looked for differences in the levels of HspBP1 in the nucleus of normal breast tissues versus different grades of breast cancer tissues using immunohistochemistry. The results show highest levels of nuclear HspBP1 in normal tissues and then progressively lower levels in human breast cancer tissues with increasing cancer grades (Fig. 8A, B), suggesting that nuclear HspBP1 expression correlates negatively with advancing clinical stages of breast tumors.

## Discussion

In the present study, we show that HspBP1 has anti-tumorigenic effects in breast cancer in a BRCA1-dependent manner. To our knowledge, this is the first report of a role for HspBP1 in tumorigenesis of breast cancer.

HspBP1 is localized to the cytoplasm, nucleus, and cell surface of tumor tissue cells [15, 33], and its expression is upregulated in a number of tumor types, including human hepatocellular [34], breast [15], and brain tumors [33]. However, HspBP1 levels inversely correlate to the aggressiveness of breast tumors [15]. Our result showed that the relative staining intensity of nuclear HspBP1 was lower in tumors than normal human breast tissues. Moreover, nuclear localization of HspBP1 negatively correlates with increasing breast cancer grades. Therefore, lower levels of HspBP1 in the nucleus may be a characteristic biological marker for breast cancers that express wild-type BRCA1.

The BRCA1 tumor suppressor is a nuclear phosphoprotein that plays a crucial role in maintaining genomic integrity [18]. BRCA1 achieves this by integrating important cellular processes such as HR-mediated DSB repair, cell cycle checkpoint control, and transcription [19, 35]. If the ability of BRCA1 to repair damaged DNA is impaired, the consequences include genomic instability, acquisition of oncogenic alterations, and ultimately tumorigenesis [36]. A novel aspect of the study described here includes the identification of HspBP1 as an important regulator of BRCA1-mediated DNA repair. We found that HspBP1 is recruited to DSBs and colocalizes with γ-H2AX in response to IR. Moreover, HspBP1 interacts with BRCA1, promoting BRCA1 phosphorylation and recruitment to DSBs following exposure to IR. Although U2OS, MCF10A, and MCF7 cells have wild-type endogenous BRCA1, the function of endogenous BRCA1 in DDR is compromised when HspBP1 is knocked down. A phenomenon termed “BRCAness” includes HR-mediated DSB repair, an error-free repair mechanism [36]. Under such conditions, cells might become more reliant on the error-prone NHEJ pathway. Therefore, a defect in the HR repair pathway in HspBP1-depleted cells may result in the accumulation of chromosome aberrations that cause genomic instability and/or tumorigenesis. Although we cannot exclude the possibility that HspBp1-induced tumor suppression is mediated by other interacting partners such as Hsp70, which plays a critical role in cancer initiation and progression [37], it is tempting to speculate that the positive impact of HspBP1 on BRCA1 functioning is related to anti- tumorigenesis in breast cancer.

When cells suffer from DNA damage, the DNA damage response (DDR) is initiated and fundamental decisions regarding DNA repair and apoptosis must be made [38]. Although the precise molecular mechanisms behind how the cell makes a choice between DNA repair and survival or apoptotic cell death are still not well understood, several DDR factors may contribute to not only promoting DNA repair at the damaged site but also facilitating apoptosis when there is extensive DNA damage by acting as a signal mediator to switch from survival pathways to cell death pathways. For example, Nijmegen breakage syndrome protein (NBS1), a component of the Mre11-Rad50-Nbs1 (MRN) complex, is a critical regulator of DNA damage repair and mediates this process through the N-terminal FHA and BRCT domain, but the C-terminus is required for IR-induced apoptosis [39–41]. H2AX, a sensor of DSBs, is phosphorylated at Ser139 in response to DNA damage, initiating the DDR [42], but phosphorylation of a carboxy-terminal tyrosine (Y142) promotes recruitment of pro-apoptotic factors, leading to apoptotic cell death [43]. In the present study, we found that HspBP1 suppresses breast cancer tumorigenesis and promotes HR-mediated DNA repair, and these effects depend on BRCA1. But, at the same time, HspBP1 promotes apoptosis in response to IR, which is independent of BRCA1. Hsp70 is powerful anti-apoptotic protein that directly interacts with Apaf-1, inhibiting the recruitment of procaspase-9 to the apoptosome, thereby suppressing apoptosis [29–31]. We found that upon IR treatment, Hsp70 strongly interacts with Apaf-1 in HspBP1-depleted cells, initiating cytochrome C release and caspase activation, consequently promoting apoptotic cell death. Importantly, upon IR treatment, HspBP1-induced cell death occurs in both BRCA1-proficient and deficient cells, suggesting that this process is independent of BRCA1. When DNA damage is so extensive that DNA repair capacity is exceeded, the apoptosis signaling pathway is activated to remove these severely damaged cells, an important mechanism that inhibits cellular transformation and immortalization. Therefore, in breast cancer patients with reduced HspBP1 expression, DNA damage accumulates due to suppressed BRCA1 function, and the ability to remove these damaged cells is decreased as a result of suppressed Hsp70 proapoptotic functions which may synergistically trigger breast cancer progression.

In summary, we provide evidence that HspBP1 plays an important role in preventing tumorigenesis in BRCA1-proficient breast cancer. Because HspBP1 interacts with BRCA1, depletion of HspBP1 results in a decrease in BRCA1 and Rad51 foci formation and impaired HR, thereby leading to an increase in chromosomal aberrations and genomic instability and driving neoplastic transformation. Notably, when a HspBP1 depletion prevents Hsp70 proapoptotic functions, this could promote the survival of these damaged cells. Thus, we propose that HspBP1 has dual functions in maintaining genomic stability and promoting apoptosis (Fig. 8C), and HspBP1 expression levels in breast cancer cells could be an important predictor of the likelihood of whether the cancer will respond to radiation.

## Supplementary information

Supplementary Figure and Table

Original Data File

Original Data File

aj-Checklist

## Data Availability

All data needed to evaluate the conclusions in the paper are present in the paper. Additional data related to this paper may be requested from the corresponding author.
