# Supplementary material for: HspBP1 is a dual function regulatory protein that controls both DNA repair and apoptosis in breast cancer cells
Source: Cell Death Dis. 2022 Apr 6;13(4):309. doi: 10.1038/s41419-022-04766-0 (PMC8986865; doi:10.1038/s41419-022-04766-0)
Supplement: Supplementary file 1 — Supplementary Figure and Table [file 41419_2022_4766_MOESM1_ESM.docx]

HspBP1

β-actin

MDA-MB-436

MCF-7

MDA-MB-231

Control shRNA

HspBP1 shRNA

Mock

Control shRNA

HspBP1 shRNA

Mock

Control shRNA

HspBP1 shRNA

Mock


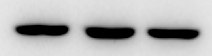

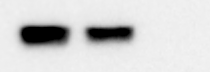

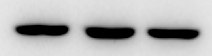

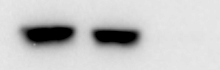

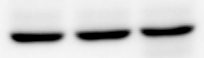

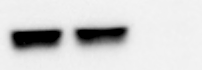


**A**

GFP-HspBP1

β-actin

MDA MB-436

MCF-7

MDA MB-231

Control

HspBP1

Mock

Control

HspBP1

Mock

Control

HspBP1

Mock


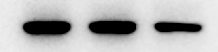

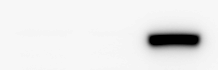

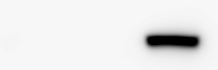

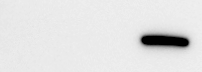

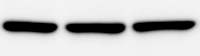

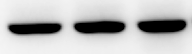


**B**

**Fig. S1 Levels of HspBP1 in breast cancer cells with HspBP1 either depleted or overexpressed. A** Immunoblot analysis of HspBP1 from stable knockdowns using HspBP1 shRNA in MCF-7, MDA-MB-231, and MDA-MB-436 cells. **B** Immunoblot analysis of HspBP1 from stable overexpression of HspBP1 in MCF-7, MDA-MB-231, and MDA-MB-436 cells.


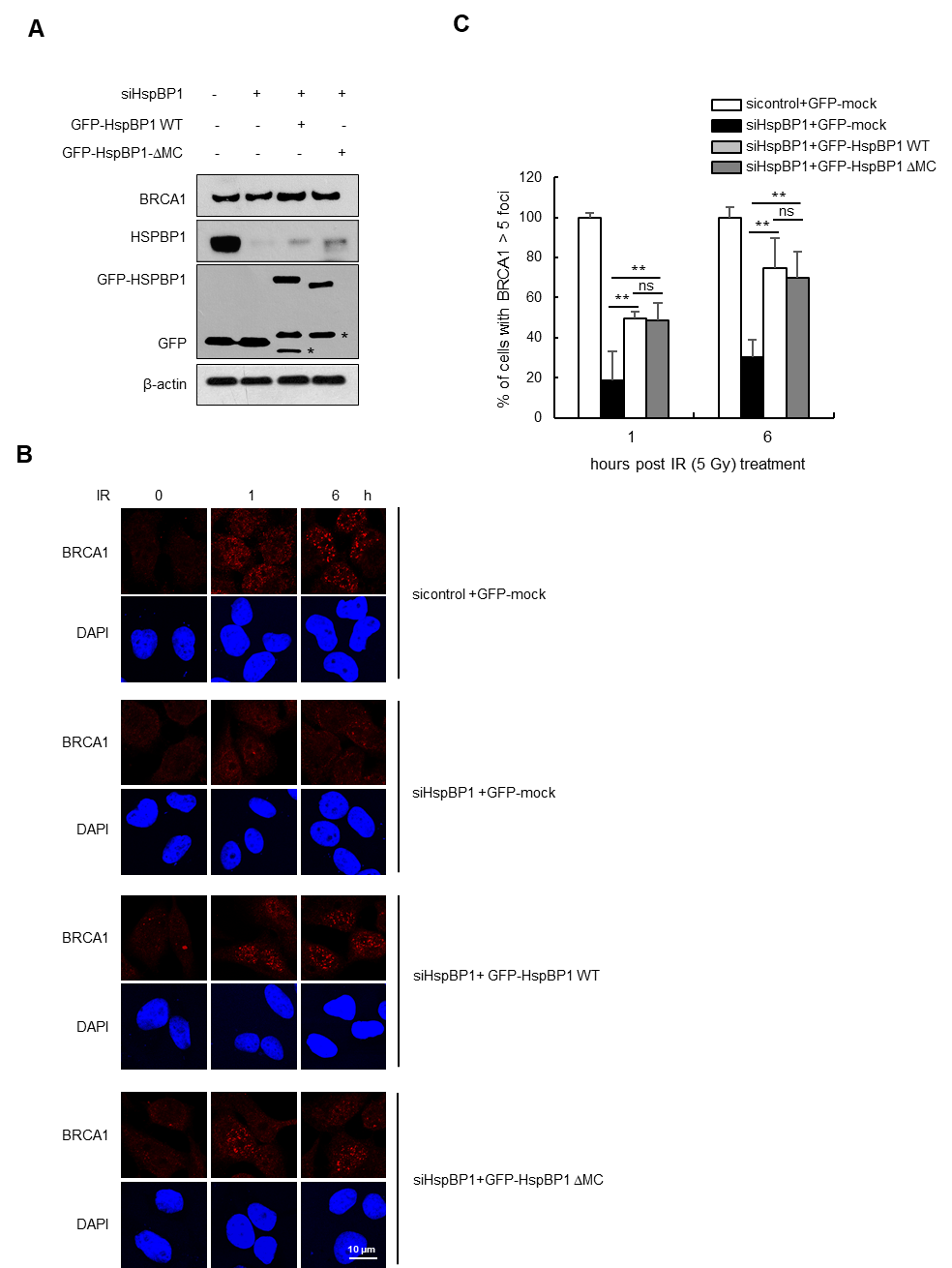


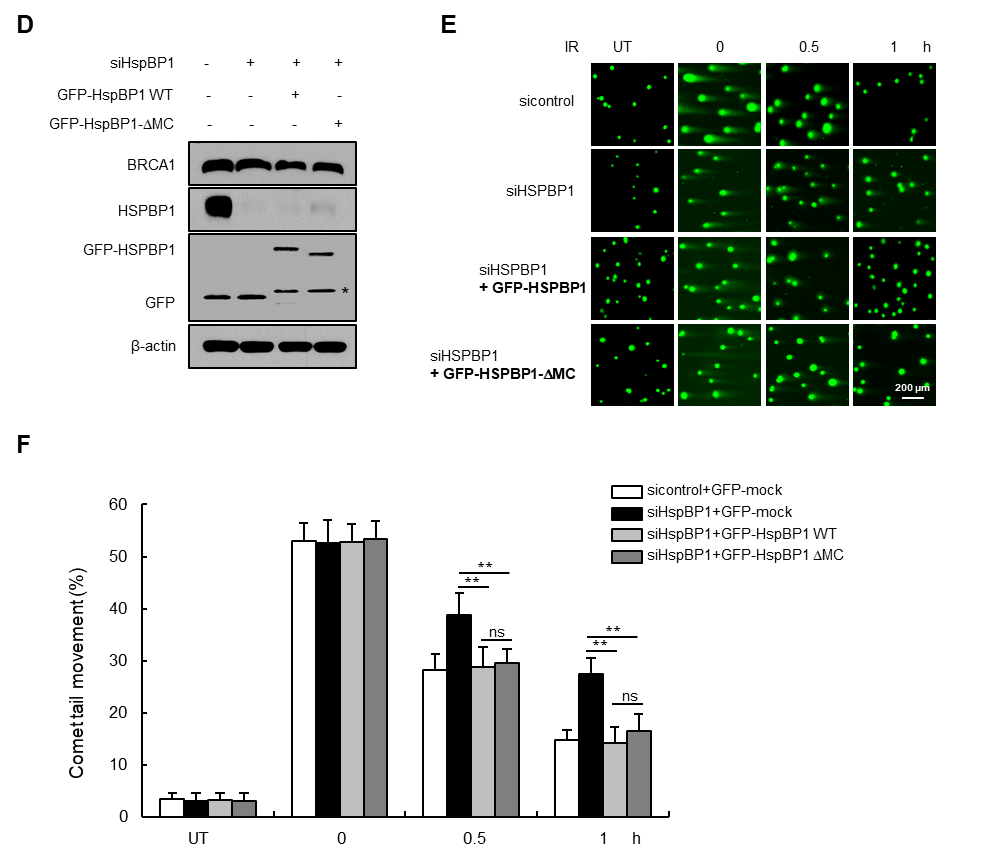


**Fig. S2 HspBP1-ΔMC rescues IR-induced BRCA1 foci and DSB repair in HspBP1-depleted HeLa cells.** **A** Immunoblot analysis of HspBP1 from HspBP1 knockdown HeLa cells reconstituted with Mock GFP, GFP-HspBP1 WT, or GFP-HspBP1-ΔMC. Asterisks indicate nonspecific bands. **B, C** HspBP1 knockdown HeLa cells reconstituted with Mock GFP, GFP-HspBP1 WT, or GFP-HspBP1-ΔMC were treated with 5 Gy of IR, fixed at 3 h, and immunostained using an anti-BRCA1 antibody. The percentage of cell populations that shows more than 10 foci for BRCA1 is shown. Representative images (**B**) and quantification of BRCA1 foci (**C**) are shown and data are presented as mean ± SD (n = 3), ** *P* < 0.01; ns, not significant, two-tailed Student’s t-test. **D.** Immunoblot analysis of HspBP1 from HspBP1 knockdown HeLa cells reconstituted with Mock GFP, GFP-HspBP1 WT, or GFP-HspBP1-ΔMC. Asterisks indicate nonspecific bands. **E, F** HspBP1 knockdown HeLa cells reconstituted with Mock GFP, GFP-HspBP1 WT, or GFP-HspBP1-ΔMC were either untreated or treated with 5 Gy of IR. At the indicated time points, cells were harvested for comet tail formation assays under neural conditions. Representative images (**E**) and quantification of unrepaired DSBs (**F**) are shown and data are presented as mean ± SD (n = 3), ** *P* < 0.01; ns, not significant, two-tailed Student’s t-test.

**
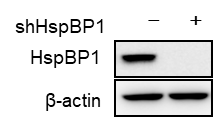
**

**A**

**B**


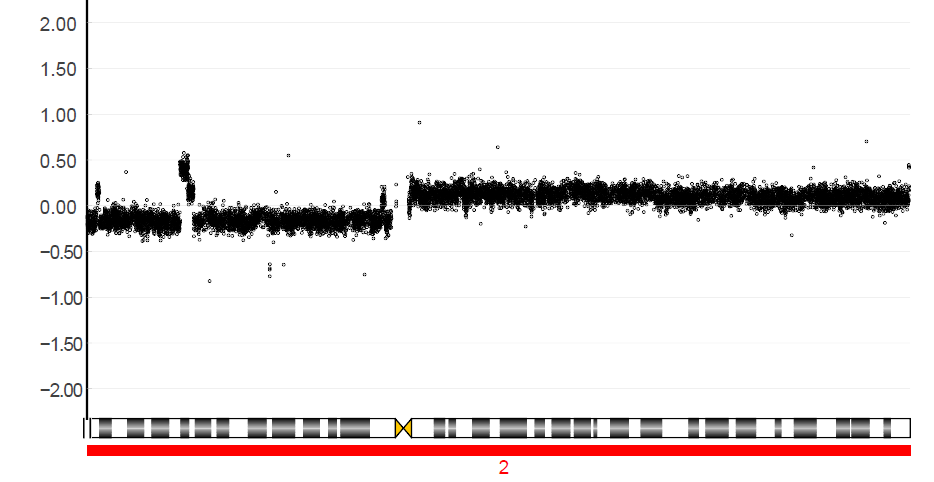

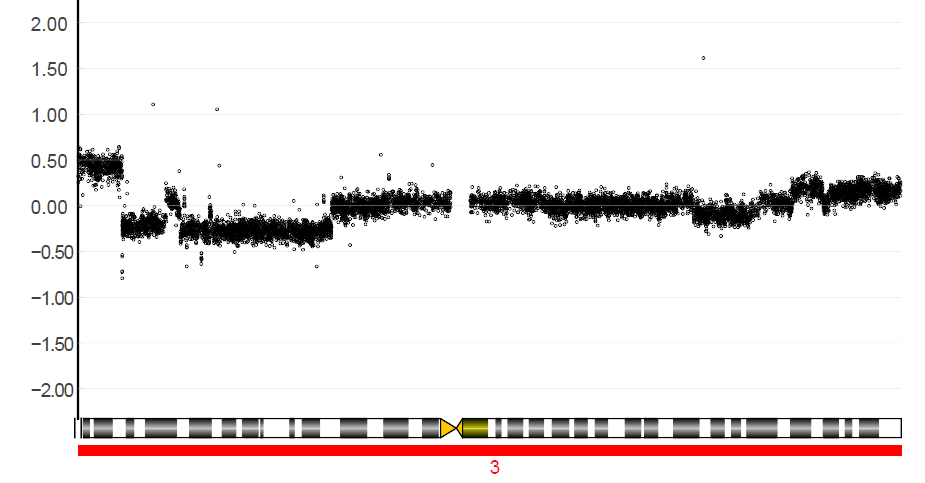

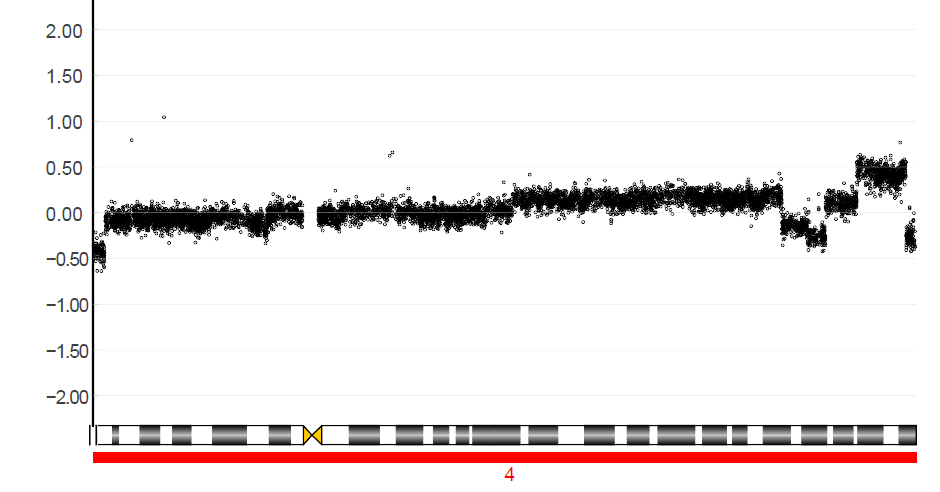

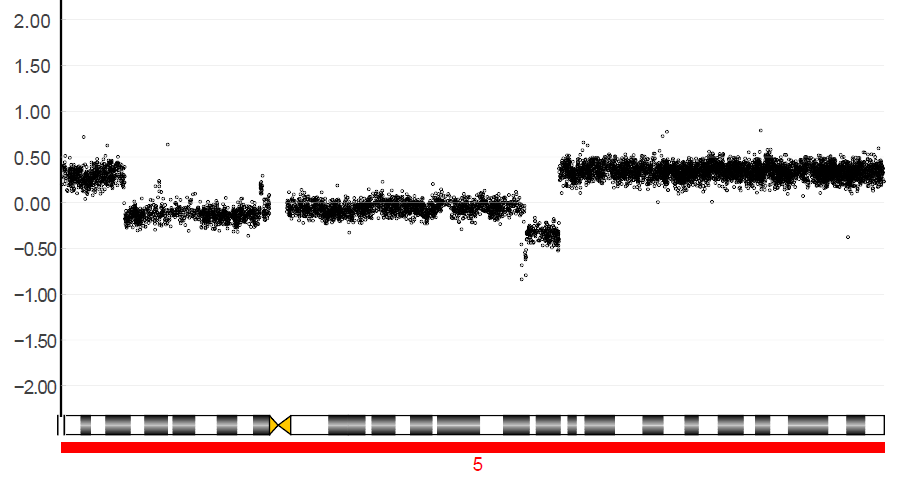

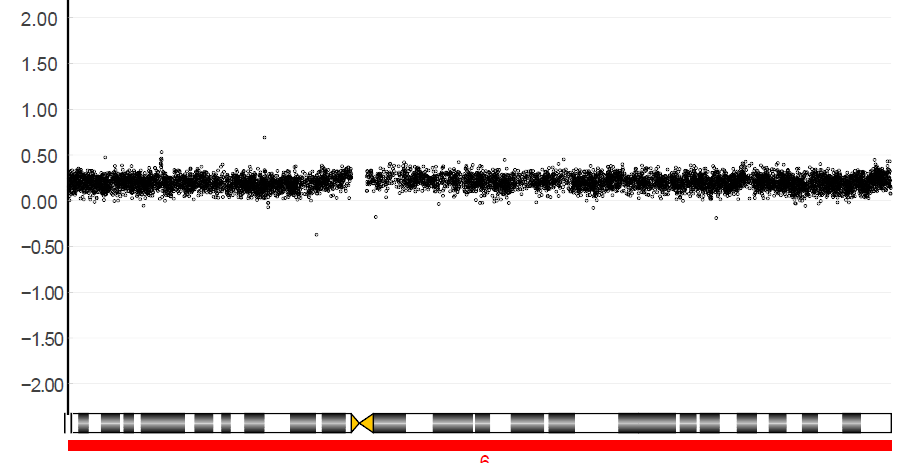

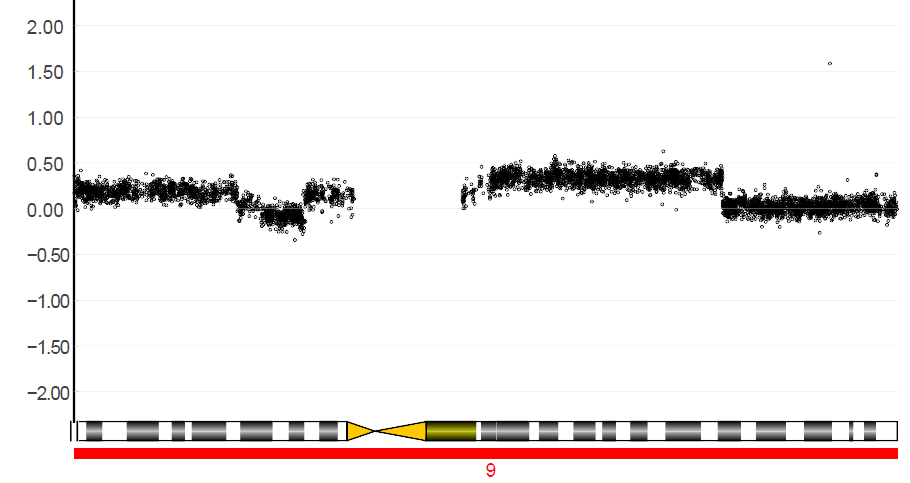

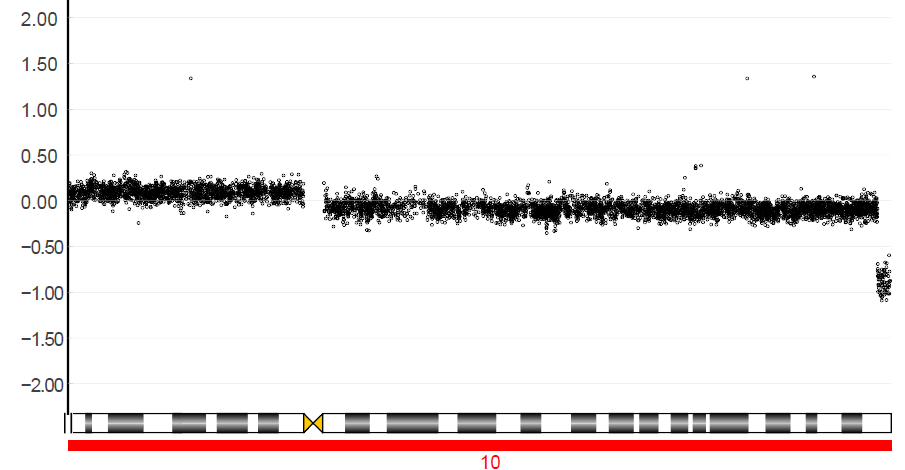

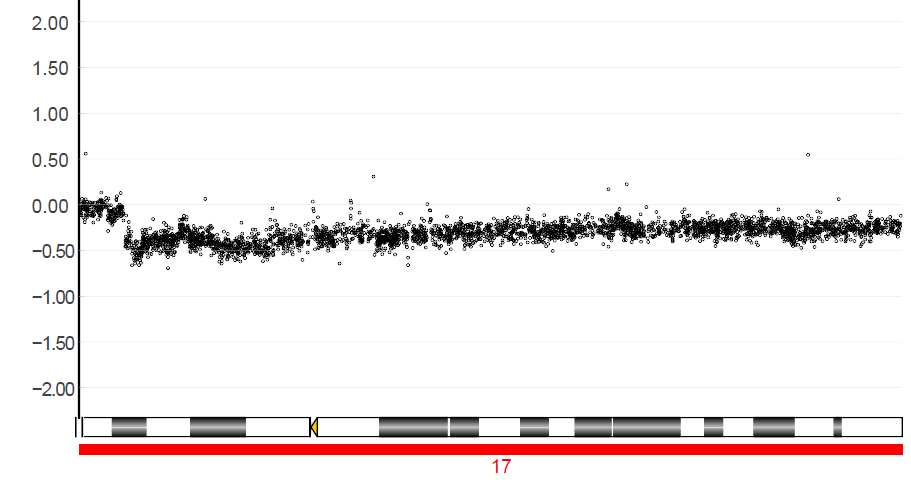

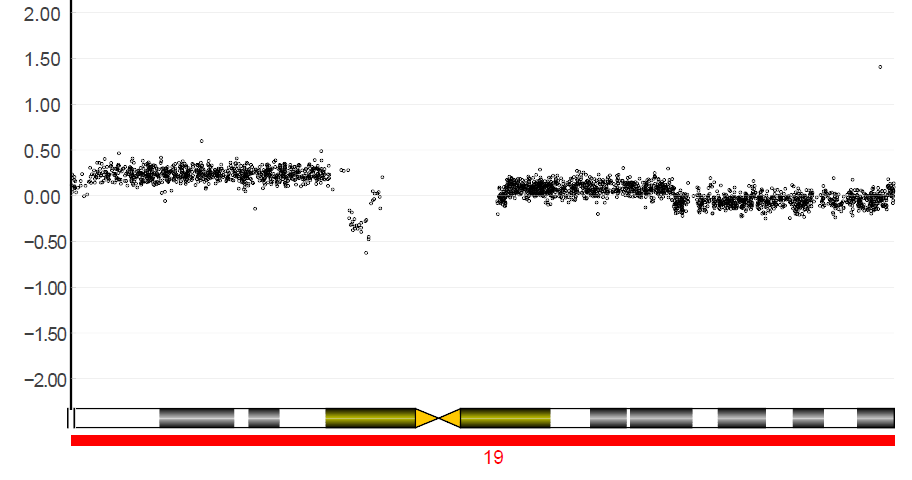


Log_2_(ratio)

Log_2_(ratio)

Chromosome 2

Chromosome 3

Chromosome 4

Chromosome 5

Chromosome 6

Log_2_(ratio)

Log_2_(ratio)

Log_2_(ratio)

Log_2_(ratio)

Chromosome 9

Chromosome 10

Chromosome 17

Log_2_(ratio)

Log_2_(ratio)

Log_2_(ratio)

Chromosome 19

**C**

shHspBP1

HspBP1

β-actin

**− +**

**
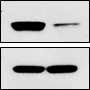
**

**D**

**
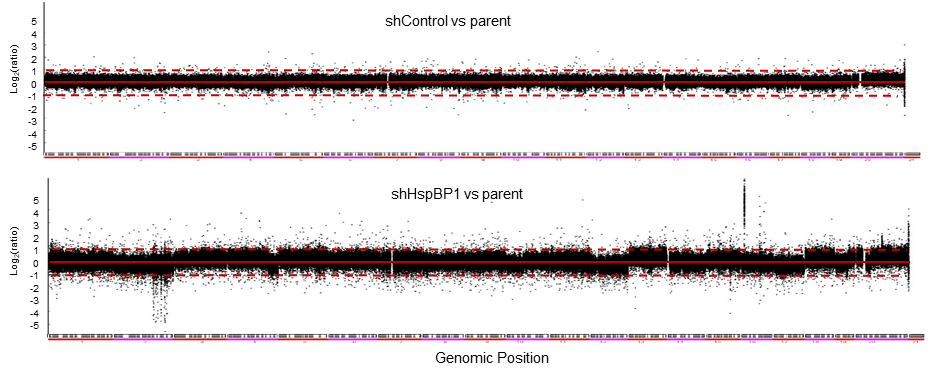
**

**Fig. S3 The effect of an HspBP1 knockdown on chromosome instability. A** Immunoblot analysis of HspBP1 from stable knockdown of HspBP1 in GM00637 cells. **B** Genome-wide DNA copy number variation in HspBP1-depleted GM00637 cells as assessed by array CGH profiles of genomic DNA derived from control and HspBP1-depleted GM00637 cells. Genomic positions that fall above or below the dotted line indicate amplifications or deletions of regions of genome, respectively. A schematic of the structure of each numbered chromosome is displayed across the X axis of each graph. **C** Immunoblot analysis of HspBP1 from stable knockdown of HspBP1 in MEFs. **D** Array CGH profiles of clones derived from control shRNA-transfected MEFs (top) and HspBP1 shRNA-transfected MEFs (bottom) are shown. Chromosomal regions above or below the dotted line indicate amplifications or deletions of genomic regions, respectively.

**
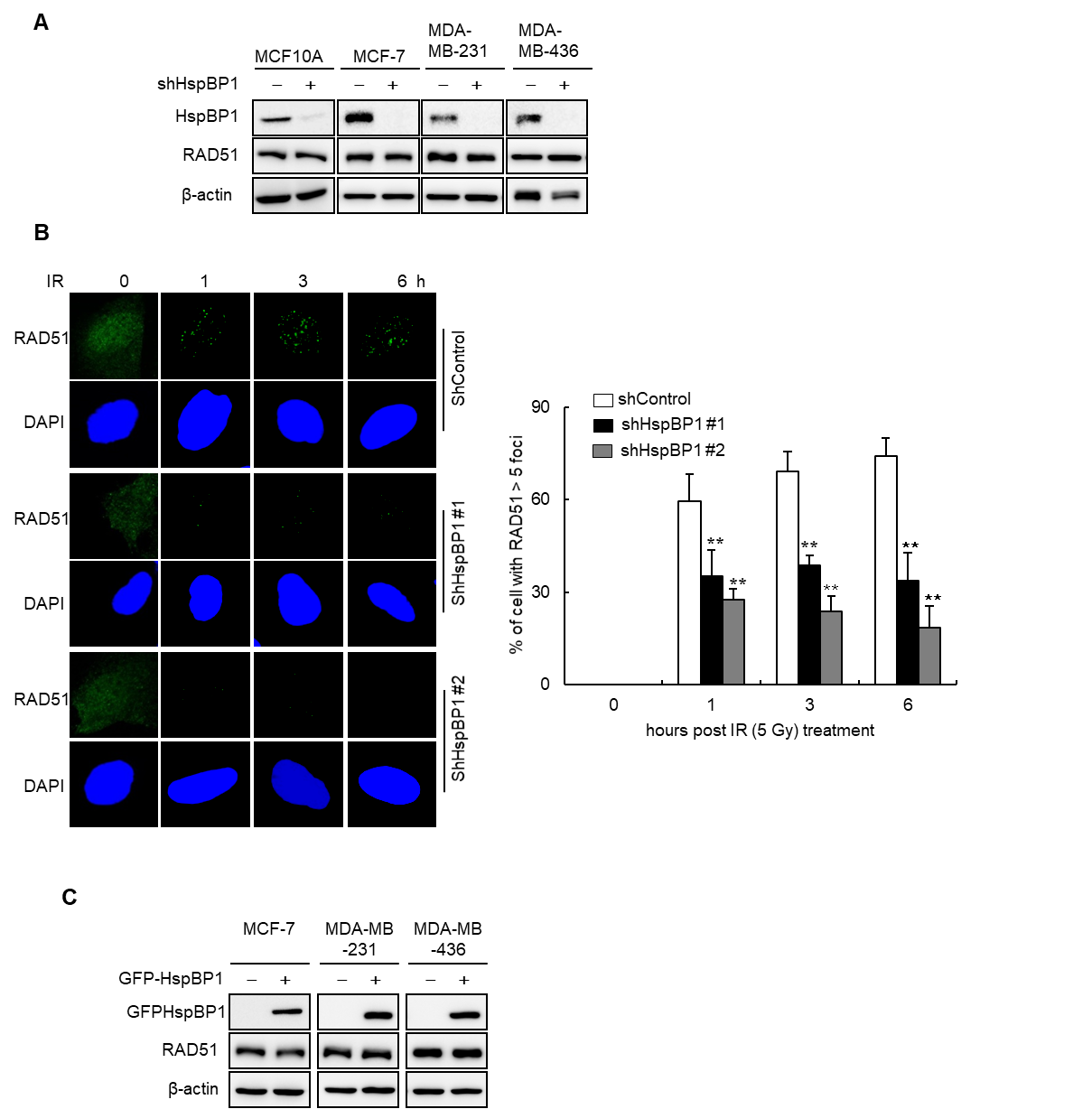
**

**Fig. S4 The level of HspBP1 in HspBP1-depleted and HspBP1-overexpressed cells. A** Immunoblot analysis of HspBP1 and Rad51 from stable knockdowns of HspBP1 in MCF-10A, MCF-7, MDA-MB-231, and MDA-MB-436 cells. **B** Control and HspBP1-depleted U2OS cells were either untreated or treated with 5 Gy IR, fixed at the indicated time points and analyzed using immunofluorescence with antibodies against Rad51. Nuclei were stained with DAPI. Representative images and the percentage of cells with more than 5 Rad51 foci are shown. Data represent mean ± SD (n = 3), ** *P* < 0.01, two-tailed Student’s t-test. **C** The levels of HspBP1 and Rad51 in control and stable GFP-HspBP1-expressing MCF7, MDC-MB-231, and MDA-MB-436 cells.

IR 5 Gy 24 h

shControl

shHspBP1

U2OS

MCF-10A

MCF-7

MDAMB-231

DAPI

MDAMB-436

DAPI

γ-H2AX


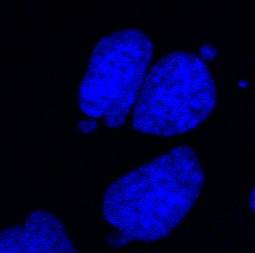

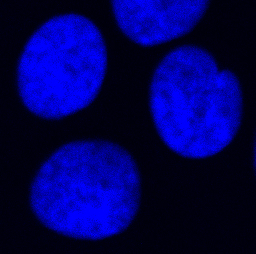

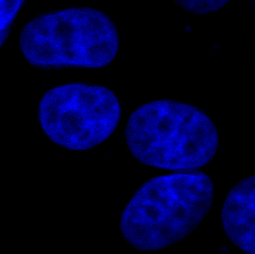

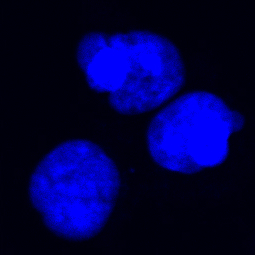

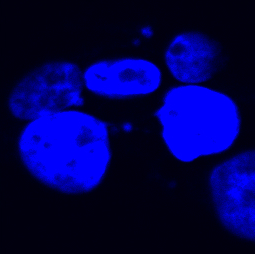

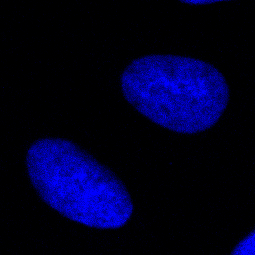

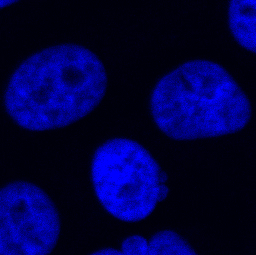

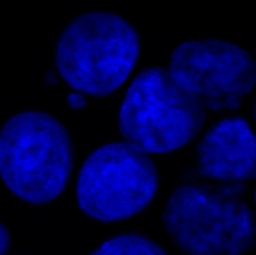

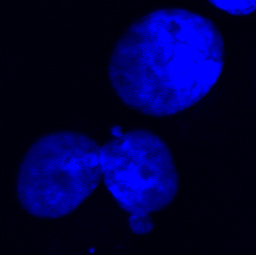

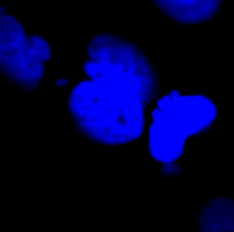

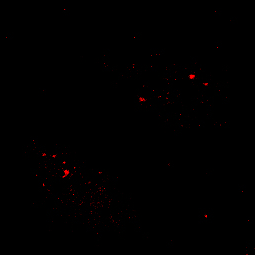

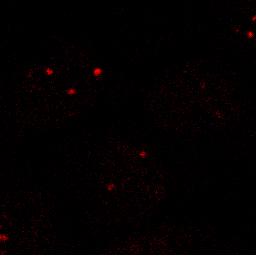

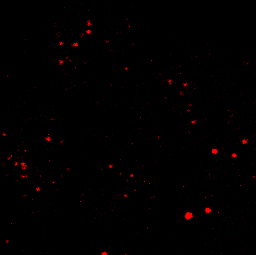

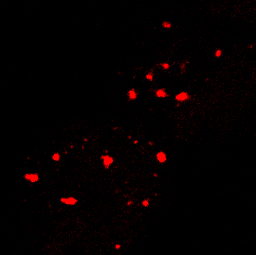

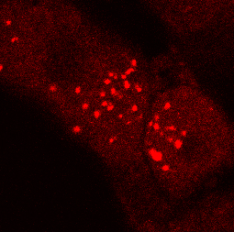

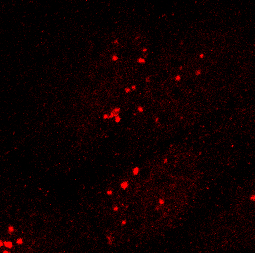

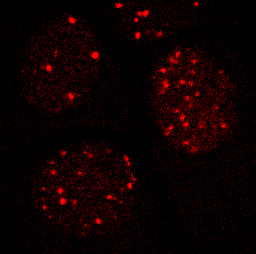

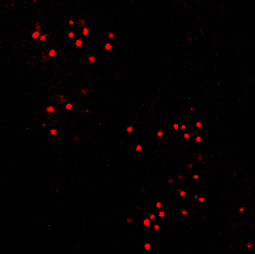

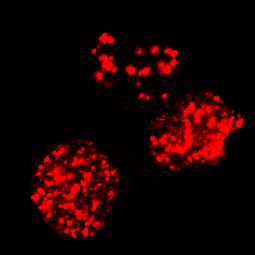

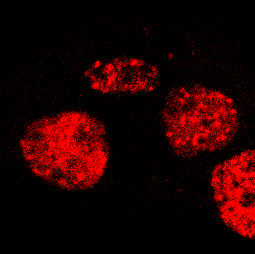


γ-H2AX

**A**

**B**

IR 5 Gy 24 h

Control -GFP

HspBP1 -GFP

MDA-MB-436

MDA-MB-231

MCF-7

DAPI

γ-H2AX

DAPI

γ-H2AX


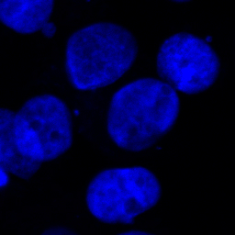

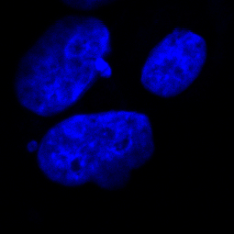

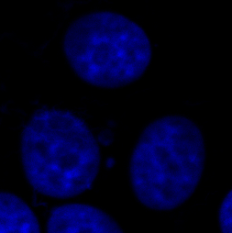

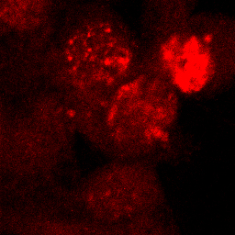

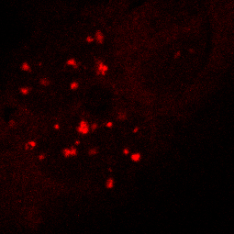

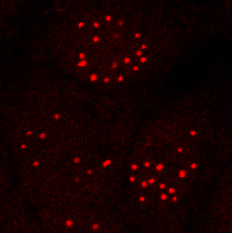

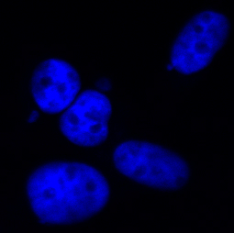

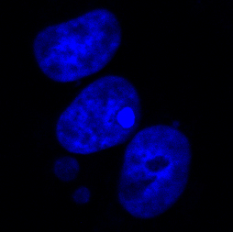

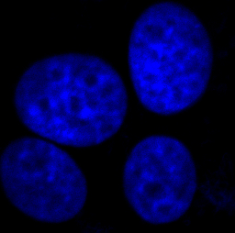

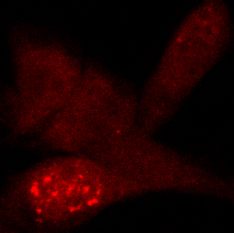

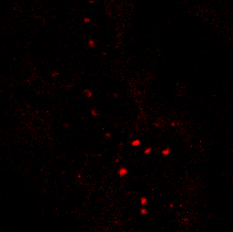

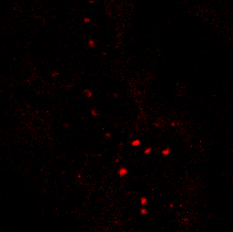

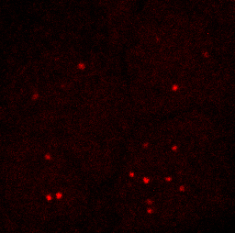

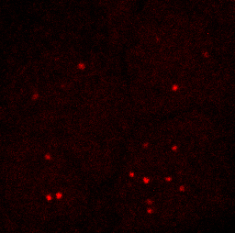


**Fig. S5 The role of HspBP1 in DSB repair. A** Control- and HspBP1-depleted U2OS, non-malignant breast cancer cells (MCF10A), BRCA1-proficient breast cancer cells (MCF7 and MDA-MB-231), and BRCA1-deficient breast cancer cells (MDA-MB-436) were treated with 5 Gy of IR, fixed after 24 h, and immunostained with antibody against γ-H2AX. Representative images are shown. DAPI staining was performed to indicate the positions of nuclei. **B** Control and HspBP1-GFP transfected MCF7, MDA-MB-231 and MDA-MB-436 cells were treated with 5 Gy of IR, fixed after 24 h, and immunostained with antibody against γ-H2AX. Representative images are shown. DAPI staining was performed to indicate the positions of nuclei.

**Table S1 List of antibodies used in this study: Western blot (WB), Immunoprecipitation (IP), Immunofluorescence (IF), and Immunohistochemistry (IHC).**

| **Antibody** | **Species** | **Application** | **Cat. No** | **Suppliers** |
| --- | --- | --- | --- | --- |
| BRCA1 | Mouse | IP | sc-6954 | Santa Cruz |
| BRCA1 | Mouse | IF | ab1524 | AbFrontier |
| BRCA1 | Rabbit | WB | 9010 | Cell signaling |
| HspBP1 | Mouse | IP, WB | sc-390467 | Santa Cruz |
| HspBP1 | Mouse | IF, IHC, WB | TA503311 | Origene |
| GFP | Rabbit | WB | sc-8334 | Santa Cruz |
| GFP | Mouse | IP | sc-9996 | Santa Cruz |
| NBS1 | Rabbit | WB | NB100-143 | Novos Biologicals |
| 53BP1 | Rabbit | WB | TA309918 | Origene |
| β-actin | Mouse | WB | ab6276 | Abcam |
| Apaf1 | Mouse | IF, WB | 611364 | BD Bioscience |
| Hsp70 | Mouse | WB | sc-24 | Santa Cruz |
| Cytochrome C | Rabbit | IF | sc-7159 | Santa Cruz |
| RAD51 | Rabbit | IF | ab63801 | Abcam |
| RAD51 | Rabbit | WB | sc-8349 | Santa Cruz |
| ATM | Rabbit | WB | LF-MA0246 | Abfrontier |
| p-BRCA1(Ser1524) | Rabbit | WB | 9009S | Cell signaling |
| p-ATM(Ser1981) | Mouse | WB | 200-301-400 | ROCKLAND |
| p-NBS1(Ser343) | Rabbit | WB | NB100-92610 | Novos Biologicals |
| p-53BP1(Ser1778) | Rabbit | WB | 2675S | Cell signaling |
| γ-H2AX | Mouse | IF | 05-636-1 | Millipore |
| HA | Rabbit | WB | sc-805 | Santa Cruz |
| HSP70 | Rabbit | WB | Sc-24 | Santa Cruz |
| HA | Mouse | IP | sc-7392 | Santa Cruz |
| Lamin A/C (sc-7292) | Mouse | WB | sc-7292 | Santa Cruz |
| α-tubulin | Mouse | WB | LF-MA0117 | Abfrontier |
| GAPDH | Goat | WB | sc-20357 | Santa Cruz |
| PARP | Mouse | WB | sc-8007 | Santa Cruz |
| Cleaved-PARP | Rabbit | WB | 5625 | Cell signaling |
| Cleaved-Caspase 9 | Rabbit | WB | 20750 | Cell signaling |
| Cleaved-Caspase 7 | Rabbit | WB | 8438 | Cell signaling |
| Donky Anti-Rabbit IgG | chicken | WB | 711-035-152 | Jackson Immunoresearch |
| Donky Anti-Mouse IgG | chicken | WB | 715-035-150 | Jackson Immunoresearch |
| Donky Anti-Goat IgG | chicken | WB | 705-035-003 | Jackson Immunoresearch |
| Alexa Fluor 488 Chicken Anti-Rabbit IgG(H+L) | chicken | IF | A-21441 | Invitrogen |
| Alexa Fluor 647 Chicken Anti-mouse IgG(H+L) | chicken | IF | A-21463 | Invitrogen |
