# Supplementary material for: HspBP1 is a dual function regulatory protein that controls both DNA repair and apoptosis in breast cancer cells
Source: Cell Death Dis. 2022 Apr 6;13(4):309. doi: 10.1038/s41419-022-04766-0 (PMC8986865; doi:10.1038/s41419-022-04766-0)
Supplement: Supplementary file 2 — Original Data File [file 41419_2022_4766_MOESM2_ESM.pptx]

## Slide 1
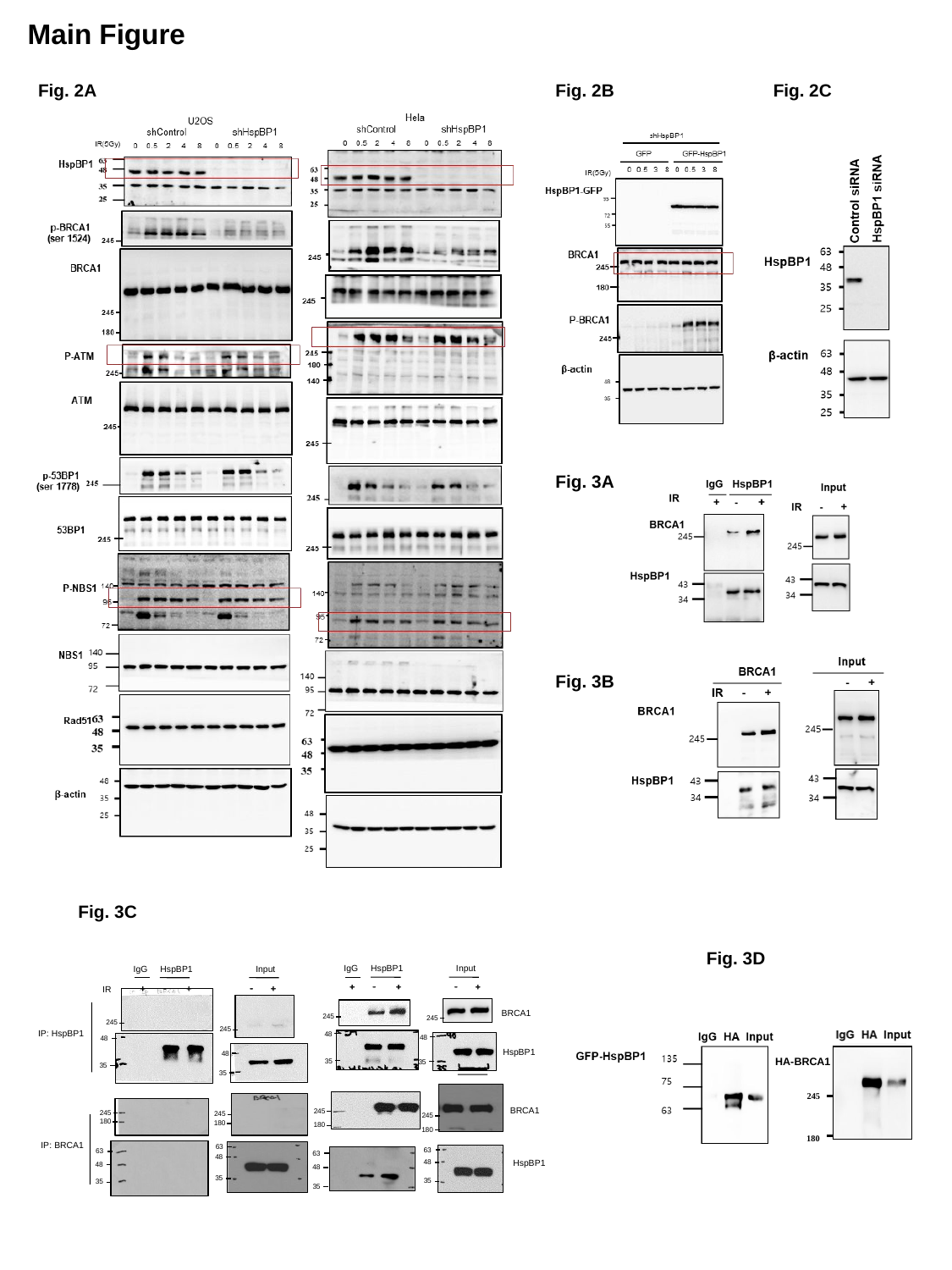

Main Figure
Fig. 2A
Fig. 2B
Fig. 2C
Fig. 3A
Fig. 3B
Fig. 3C
Fig. 3D
 IgG HspBP1 Input
 IgG HspBP1 Input
 + − + − +
 + − + − +
IR
245
48
35
BRCA1
245
245
245
IP: HspBP1
48
48
HspBP1
48
35
35
35
BRCA1
245
245
245
245
180
180
180
180
IP: BRCA1
63
63
63
63
48
48
HspBP1
48
48
35
35
35
35
HA-BRCA1
245
180

## Slide 2
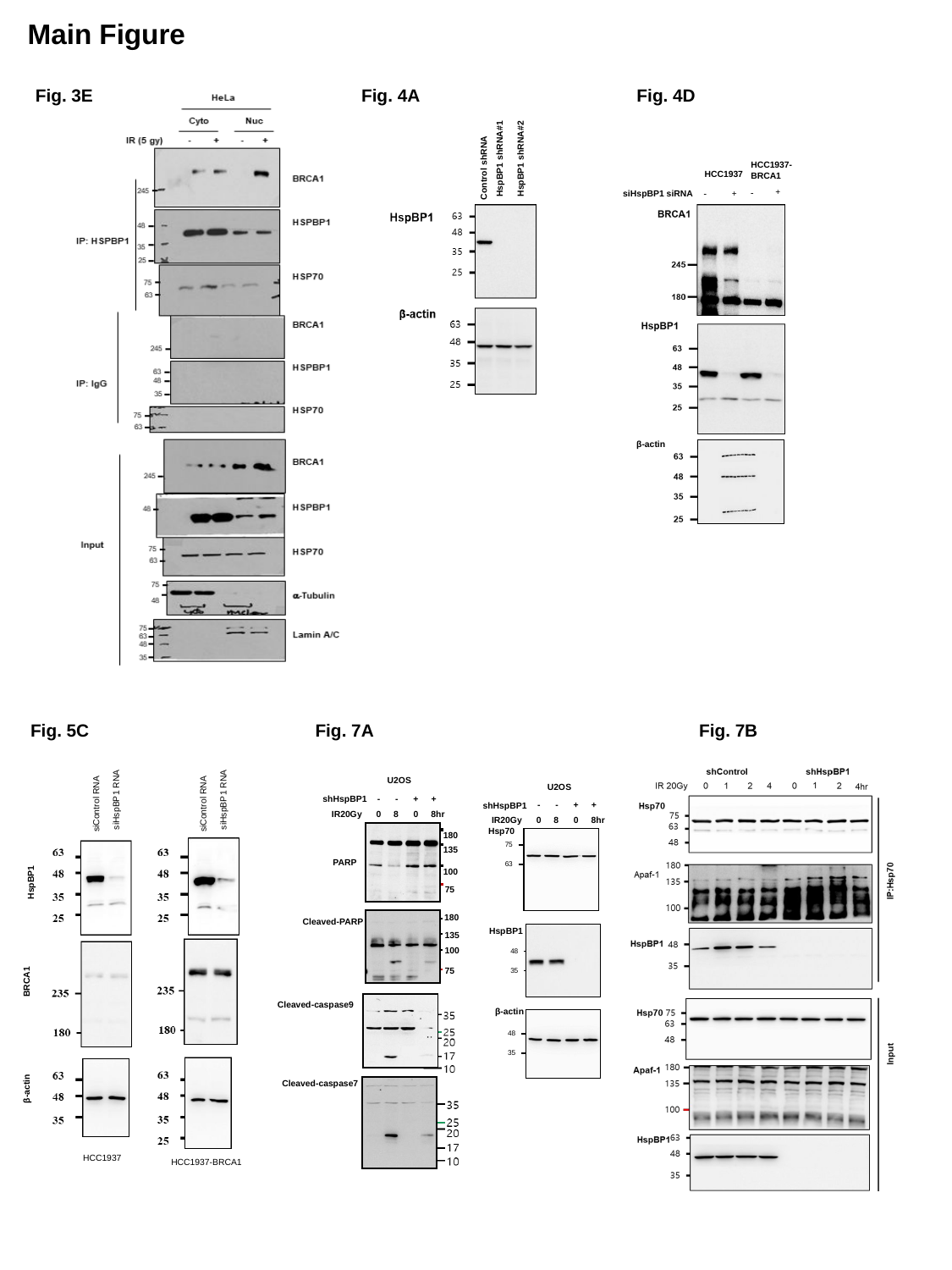

Main Figure
Fig. 3E
Fig. 4A
Fig. 4D
HspBP1 shRNA#2
HspBP1 shRNA#1
Control shRNA
HCC1937-
BRCA1
HCC1937
+
-
-
+
siHspBP1 siRNA
β-actin
Fig. 5C
Fig. 7A
Fig. 7B
siHspBP1 RNA
siControl RNA
siHspBP1 RNA
siControl RNA
HspBP1
BRCA1
β-actin
HCC1937
HCC1937-BRCA1
U2OS
U2OS
- - + +
shHspBP1
IR20Gy
0 8 0 8hr
Hsp70
75
63
HspBP1
48
35
β-actin
48
35
- - + +
shHspBP1
IR20Gy
0 8 0 8hr
180
135
PARP
100
75
180
Cleaved-PARP
135
100
75
Cleaved-caspase9
Cleaved-caspase7

## Slide 3
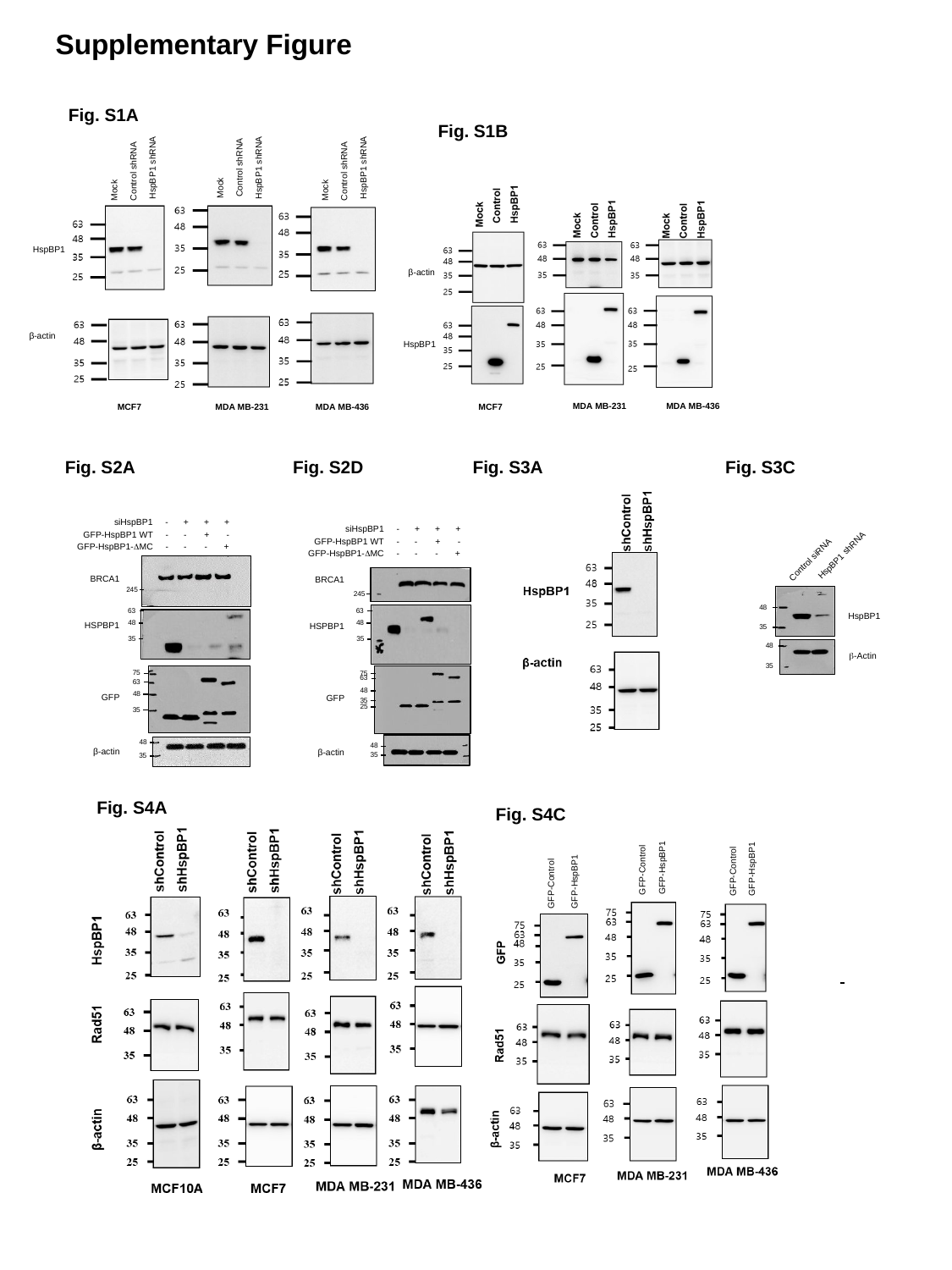

Supplementary Figure
Fig. S1A
Fig. S1B
HspBP1 shRNA
HspBP1 shRNA
HspBP1 shRNA
Control shRNA
Control shRNA
Control shRNA
Mock
Mock
Mock
HspBP1
β-actin
MCF7
MDA MB-231
MDA MB-436
β-actin
HspBP1
MDA MB-231
MDA MB-436
MCF7
Fig. S2A
Fig. S2D
Fig. S3A
Fig. S3C
siHspBP1 - + + +
GFP-HspBP1 WT - - + -
GFP-HspBP1-ΔMC - - - +
BRCA1
245
63
48
HSPBP1
35
75
63
48
GFP
35
48
β-actin
35
siHspBP1 - + + +
GFP-HspBP1 WT - - + -
GFP-HspBP1-ΔMC - - - +
BRCA1
245
63
48
HSPBP1
35
75
63
48
GFP
35
25
48
35
β-actin
HspBP1 shRNA
Control siRNA
48
HspBP1
35
48
β-Actin
35
Fig. S4A
Fig. S4C
GFP-HspBP1
GFP-HspBP1
GFP-Control
GFP-Control
GFP-HspBP1
GFP-Control
