# Supplementary material for: HspBP1 is a dual function regulatory protein that controls both DNA repair and apoptosis in breast cancer cells
Source: Cell Death Dis. 2022 Apr 6;13(4):309. doi: 10.1038/s41419-022-04766-0 (PMC8986865; doi:10.1038/s41419-022-04766-0)

Figure 1A

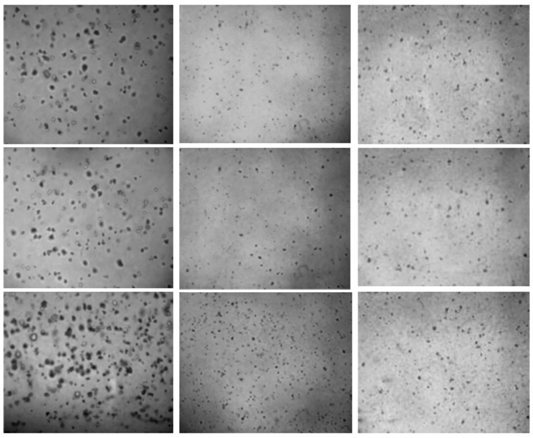

Figure 1B

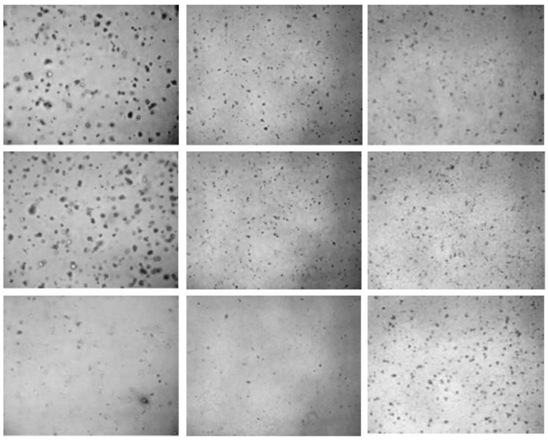

Figure 1C

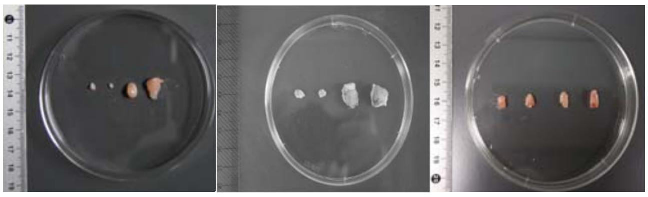

Figure 1E

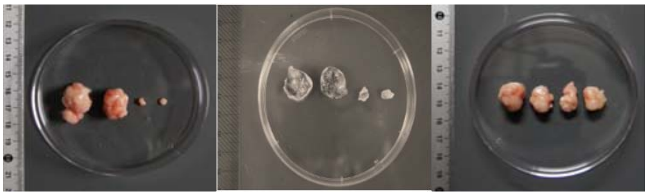

Figure 2D

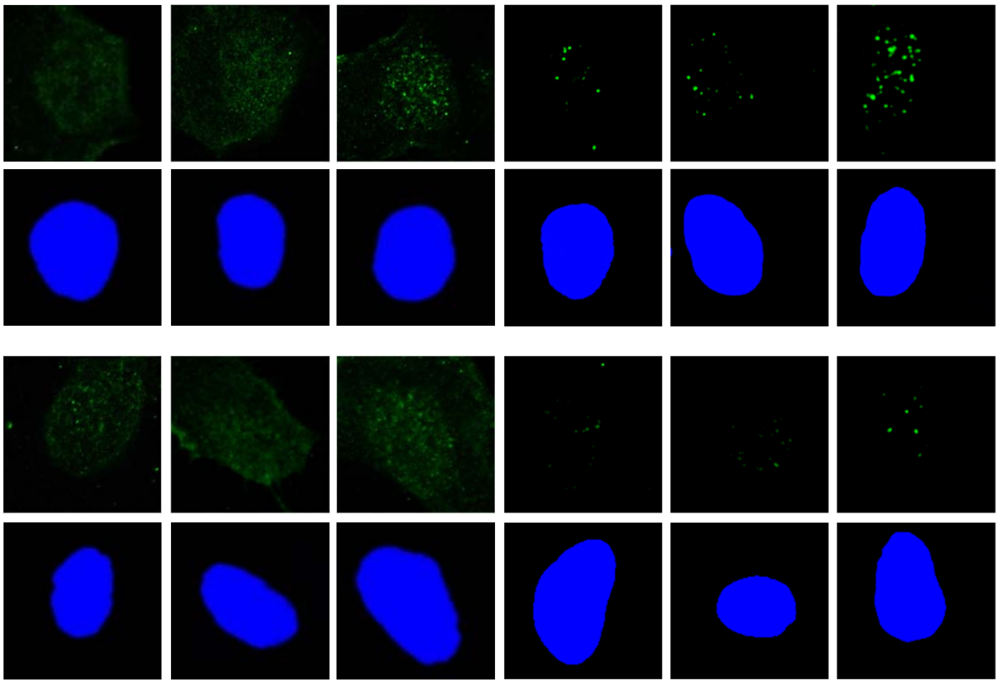

Figure 3F

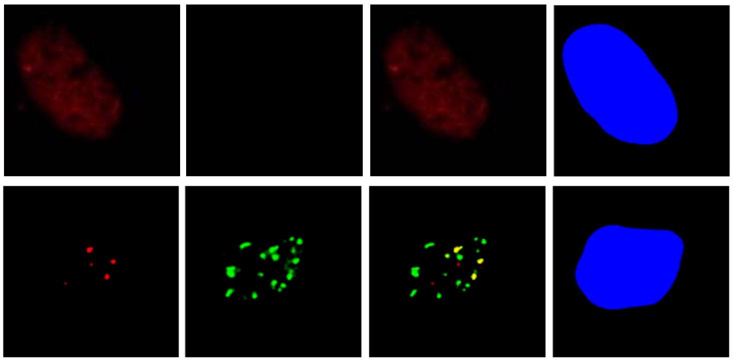

Figure4 B

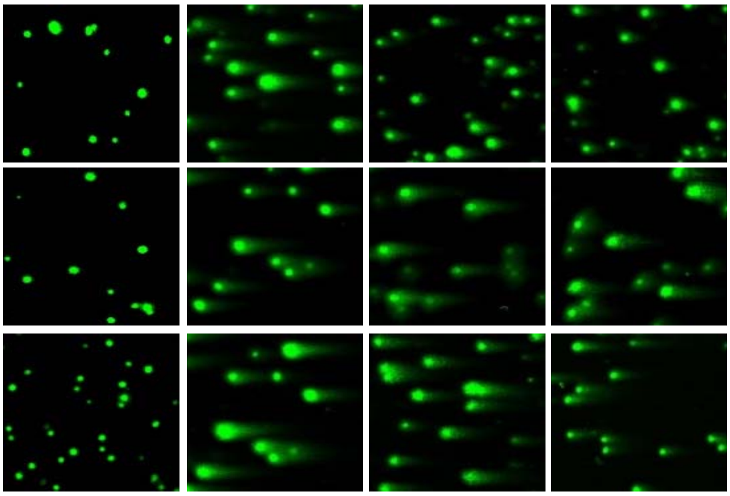

Figure 4E

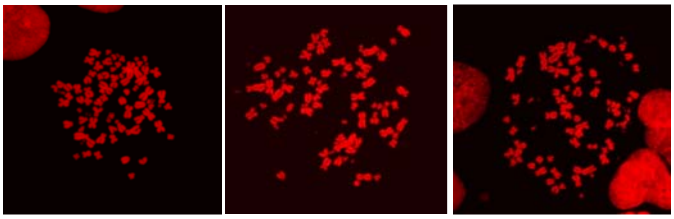

Figure5 A

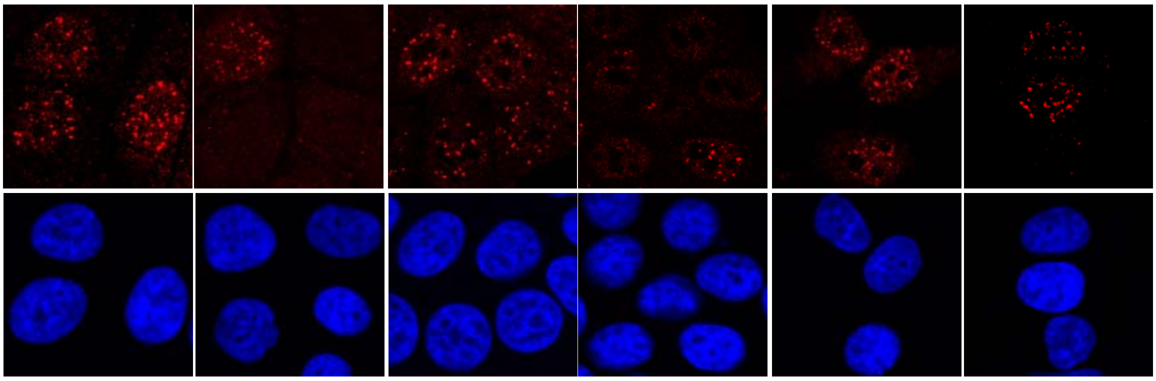

Figure5 D

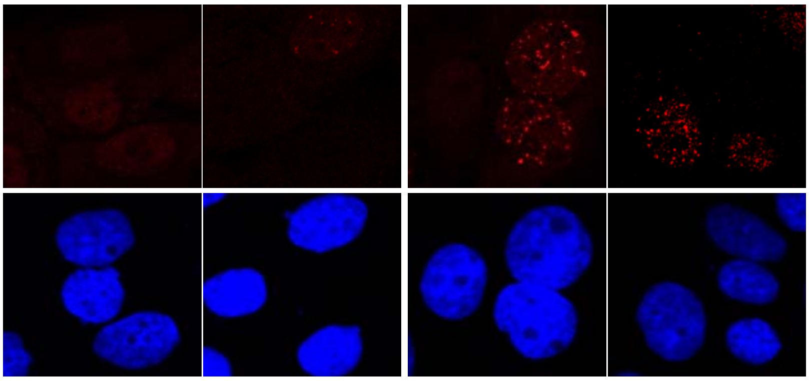

Figure5 F

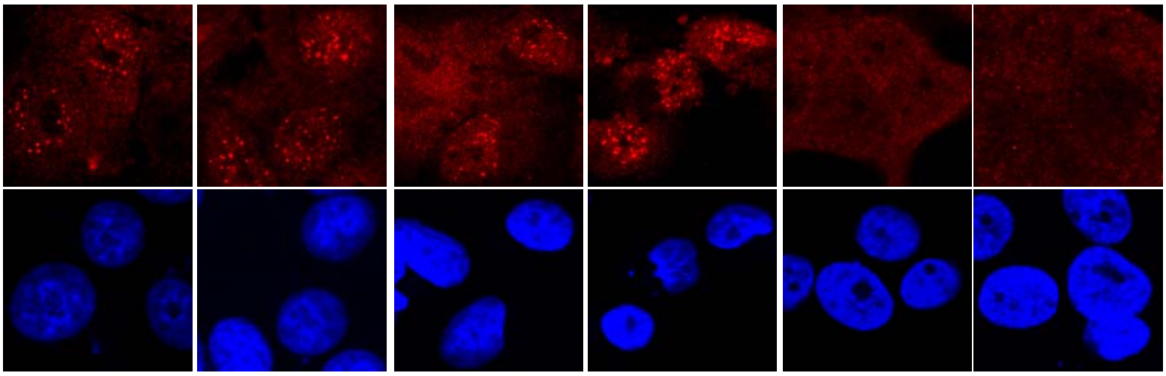

Figure 7C

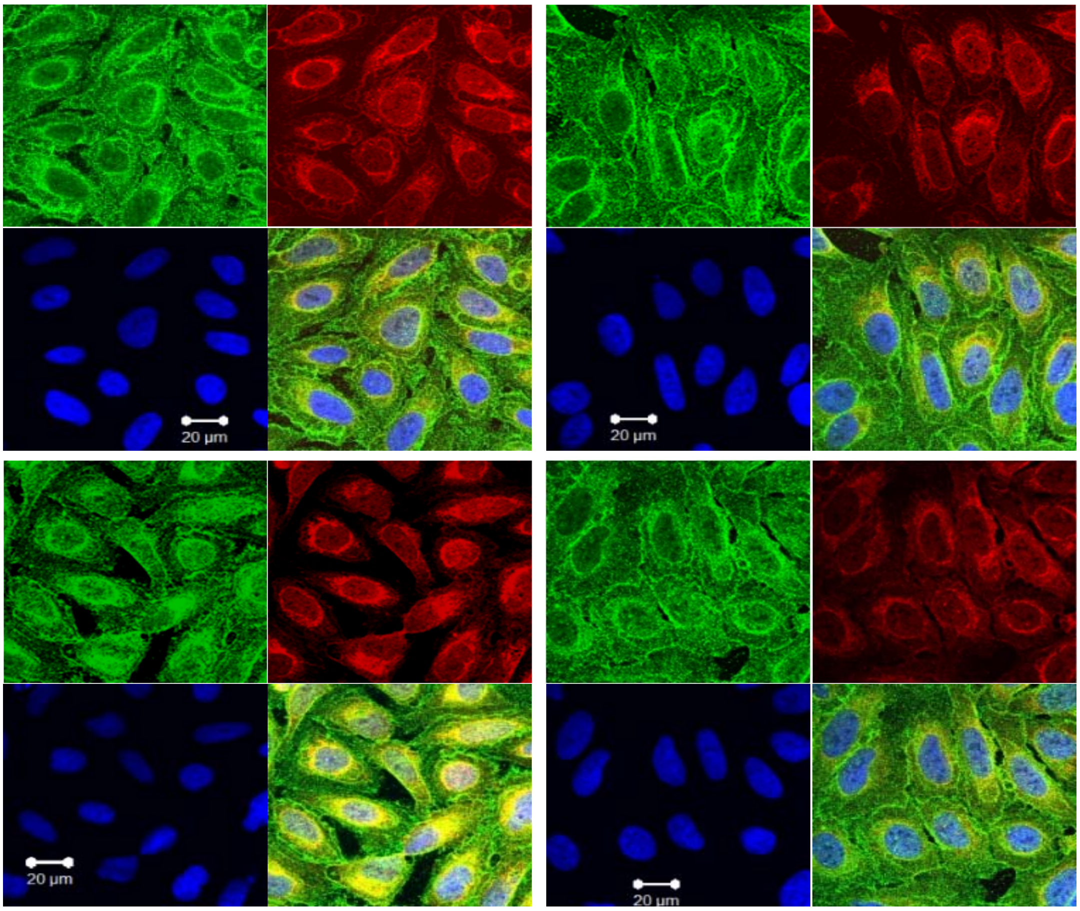

Figure 8A

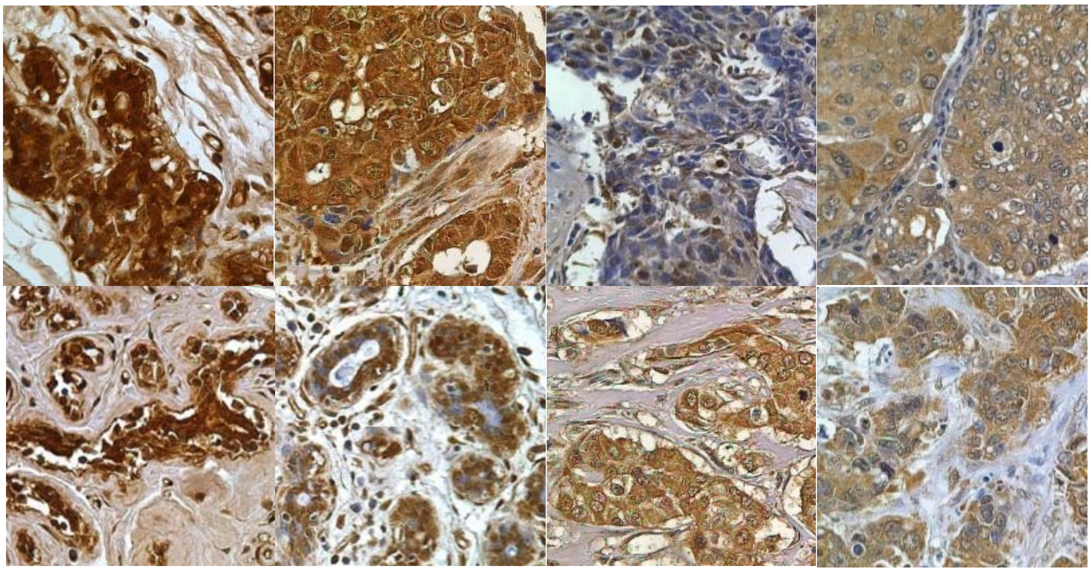

Figure S3B

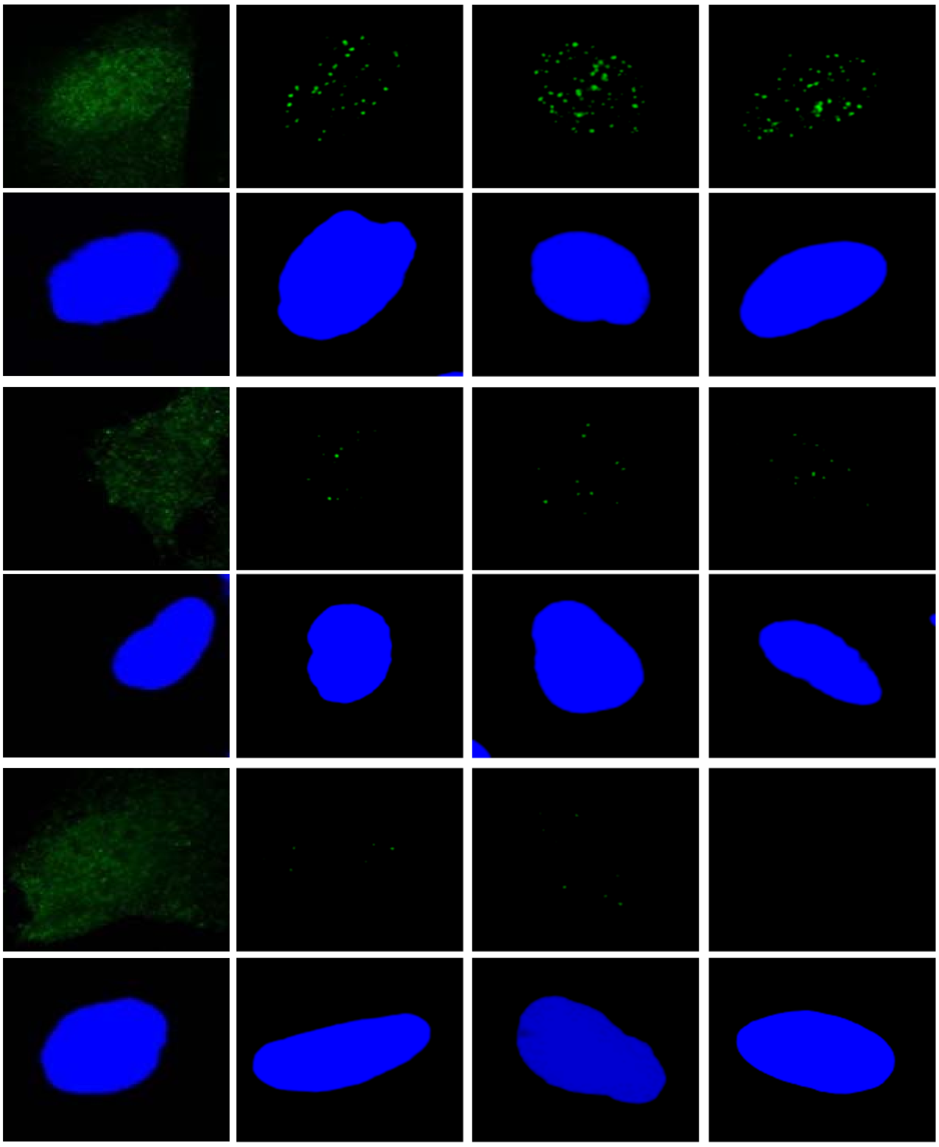

Figure S4A

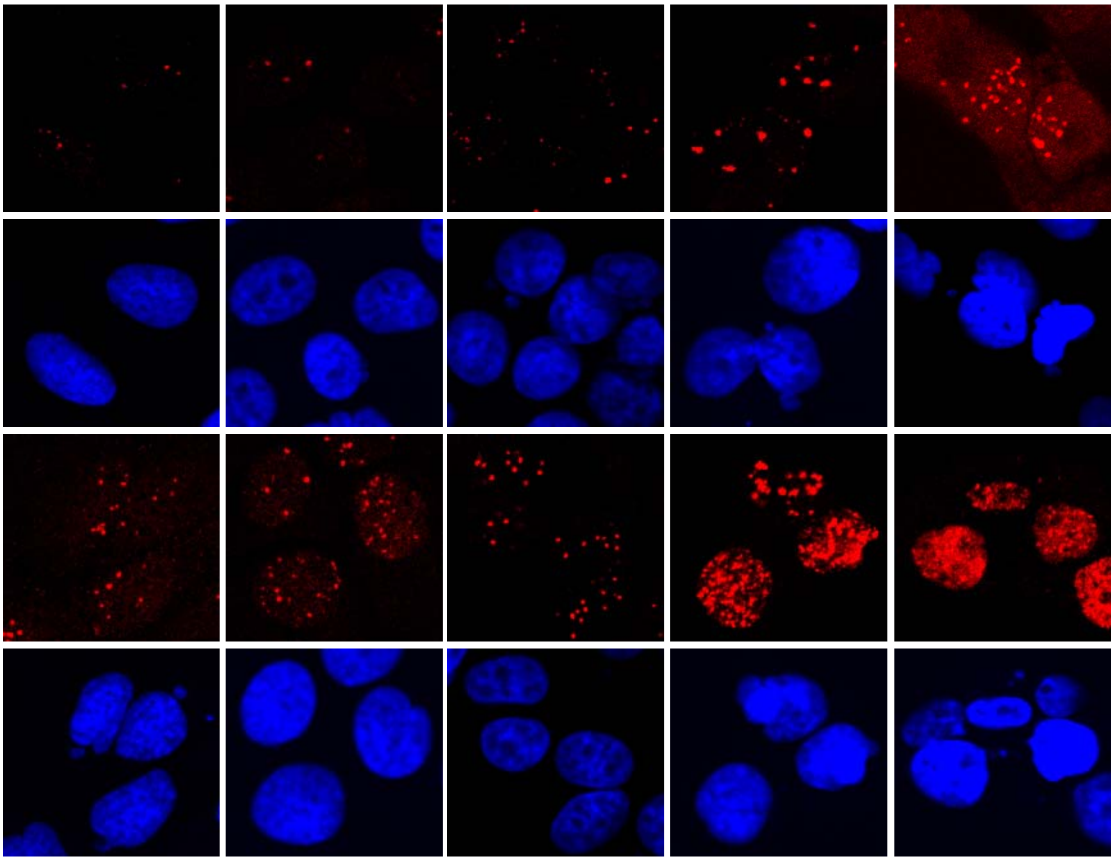

Figure S4B

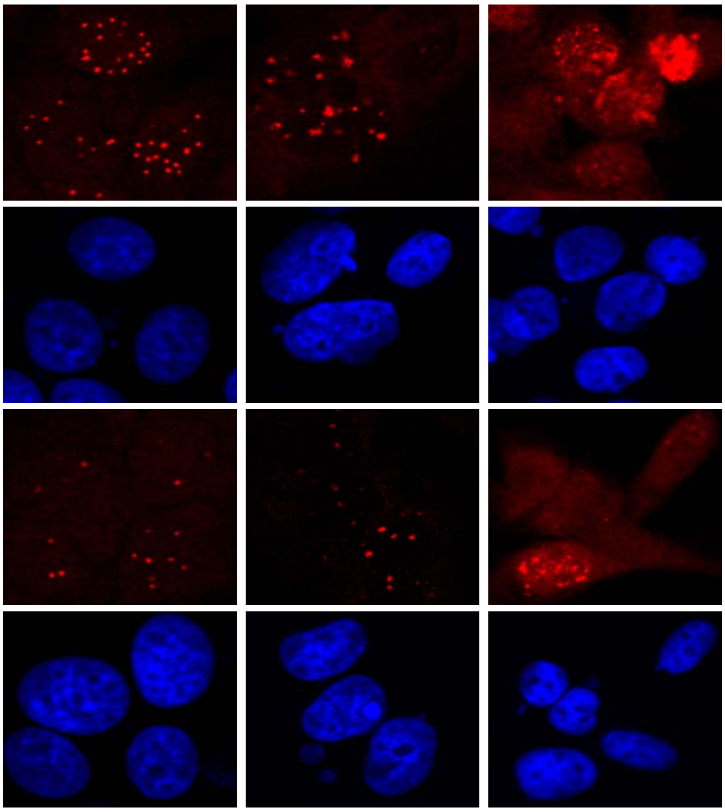

Figure S5B

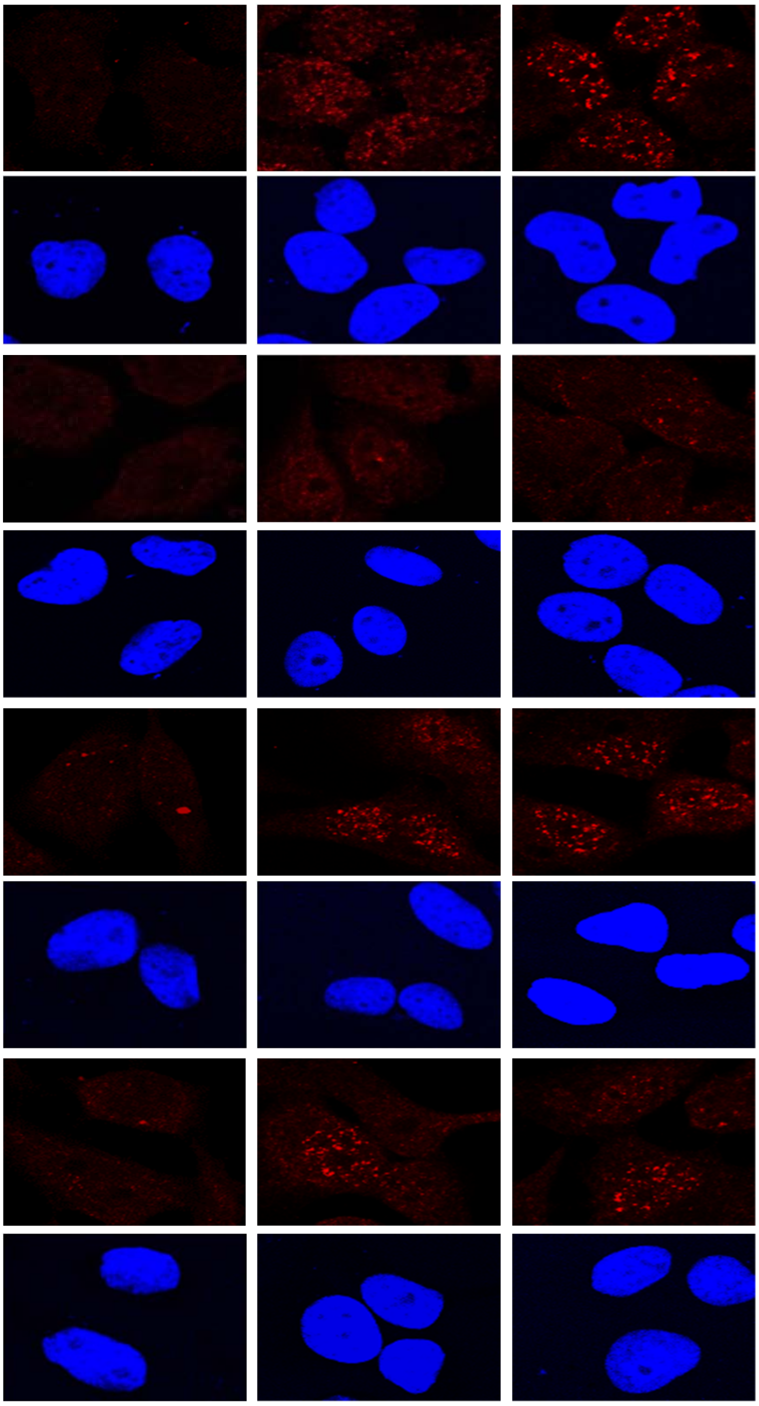

Figure S5E

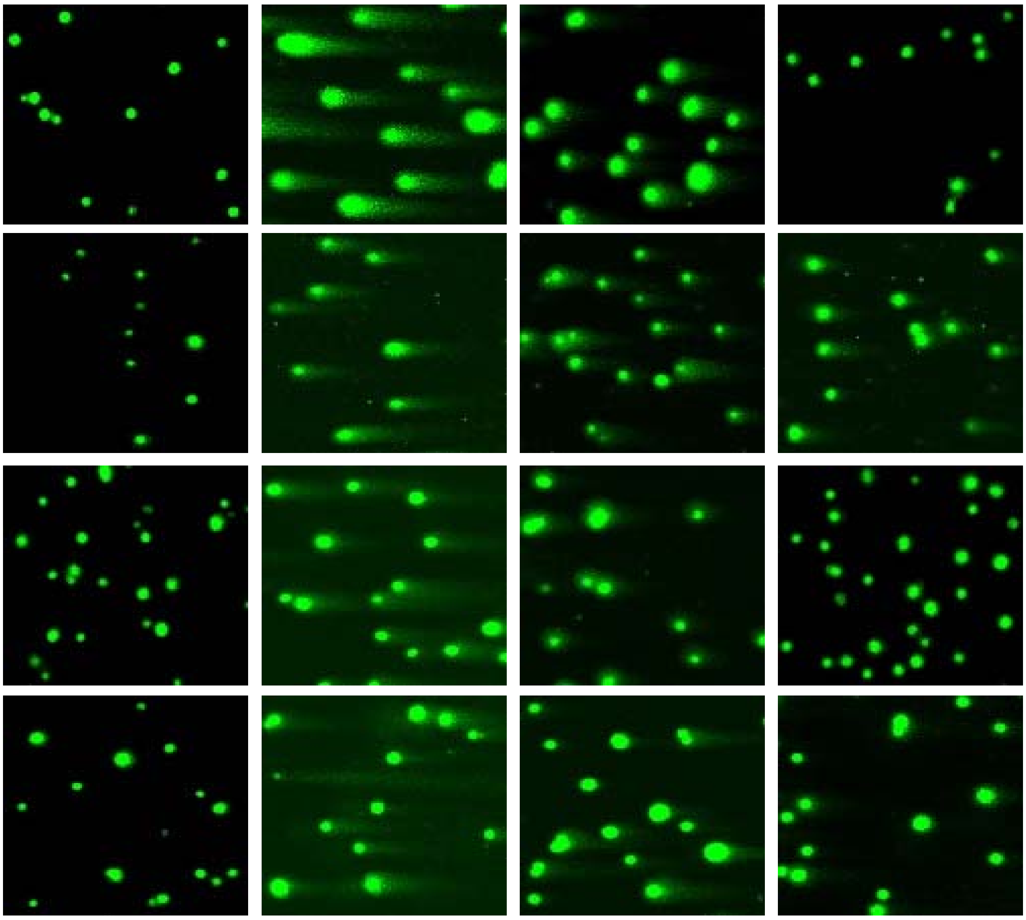

Supplement: Supplementary file 3 — Original Data File [file 41419_2022_4766_MOESM3_ESM.pdf]
